# A Self‐Constructed Mg^2+^/K^+^ Co‐Doped Prussian Blue with Superior Cycling Stability Enabled by Enhanced Coulombic Attraction

**DOI:** 10.1002/advs.202406842

**Published:** 2024-09-20

**Authors:** Zheng Xu, Fengqin Chen, Yinda Li, Yunhao Lu, Aijun Zhou, Jicheng Jiang, Xiongwen Xu, Jian Tu, Bin Pan, Fang Chen, Yi Huang, Xinbing Zhao, Jian Xie

**Affiliations:** ^1^ State Key Laboratory of Silicon and Advanced Semiconductor Materials, School of Materials Science and Engineering Zhejiang University Hangzhou 310058 China; ^2^ Shaanxi Coal Chemical Industry Technology Research Institute Xi'an 710100 China; ^3^ School of Physics Zhejiang University Hangzhou 310058 China; ^4^ Yangtze Delta Region Institute (Huzhou) University of Electronic Science and Technology of China Huzhou 313000 China; ^5^ LI‐FUN Technology Corporation Limited Zhuzhou 412000 China; ^6^ Yuna Technology Corporation Limited Hangzhou 311121 China; ^7^ Department of Chemistry Zhejiang University Hangzhou 310058 China

**Keywords:** Mg^2+^/K^+^ co‐doping, prussian blue, sea water, sodium ion batteries, sodium salts recycling

## Abstract

Prussian blue (PB) is regarded as a promising cathode for sodium‐ion batteries because of its sustainable precursor elements (e.g., Mn, Fe), easy preparation, and unique framework structure. However, the unstable structure and inherent crystal H_2_O restrain its practical application. For this purpose, a self‐constructed trace Mg^2+^/K^+^ co‐doped PB prepared via a sea‐water‐mediated method is proposed to address this problem. The Mg^2+^/K^+^ co‐doping in the Na sites of PB is permitted by both thermodynamics and kinetics factors when synthesized in sea water. The results reveal that the introduced Mg^2+^ and K^+^ are immovable in the PB lattices and can form stronger K‒N and Mg‒N Coulombic attraction to relieve phase transition and element dissolution. Besides, the Mg^2+^/K^+^ co‐doping can reduce defect and H_2_O contents. As a result, the PB prepared in sea water exhibits an extremely long cycle life (80.1% retention after 2400 cycles) and superior rate capability (90.4% capacity retention at 20 C relative to that at 0.1 C). To address its practical applications, a sodium salts recycling strategy is proposed to greatly reduce the PB production cost. This work provides a self‐constructed Mg^2+^/K^+^ co‐doped high‐performance PB at a low preparation cost for sustainable, large‐scale energy storage.

## Graphical abstract

A self‐constructed trace Mg^2+^/K^+^ co‐doped Prussian blue is prepared in sea water that exhibits extremely long cycling stability with 80.1% retention after 2400 cycles at 5C. The “pillar” effect of Mg^2+^/K^+^ and strong Mg‒N and K‒N Coulombic attraction well stabilize structure, and alleviate phase transform and elements dissolution. A low‐cost and sustainable route is provided by recycling Na salts.

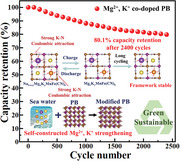

## 1 Introduction

Sodium ion batteries are considered as the next generation electrochemical devices for large‐scale energy storage because of low cost, abundant resources, environmental friendliness, and high safety.^[^ ^1^, ^2^, ^3^, ^4^, ^5^ ^]^ The performance of the cathode materials plays a key role in realizing a stable and high‐efficiency energy storage. Among various cathode materials, Prussian blue (PB) materials with a rigid and open structure that ensures rapid Na^+^ mobility and adaptive volume variation have received enormous interests and in recent years, there are some companies and researchers focusing on the PB technology.^[^ ^6^, ^7^, ^8^, ^9^, ^10^ ^]^ The general formula of Na‐based PB can be described as Na_x_M_1_[Fe(CN)_6_]_1−y_□_y_·nH_2_O, where M_1_ are the transition metals and □ represents the [Fe(CN)_6_] vacancy (0 ≤ *x* ≤ 2, 0 ≤ *y* < 1).^[^ ^11^, ^12^, ^13^ ^]^ As previously reported, the rational selection of transition metals in M_1_ site can realize either high energy density (M_1_ = Mn) or long cycle life (M_1_ = Ni, Fe), showing a great potential in large‐scale energy storage.^[^ ^14^, ^15^, ^16^, ^17^, ^18^, ^19^ ^]^

Recently, Na site doping strategy with trace alkali metal ions and alkali earth metal ions was reported to effectively improve electrochemical performance of PB by reducing H_2_O content to stabilize the framework structure and to facilitate Na^+^ migration.^[^ ^20^, ^21^ ^]^ However, for large‐scale preparation of PB, besides electrochemical performance, rational selections of precursors and preparation routes should also be taken into consideration. It is noted that the preparation of PB would consume a lot of deionized (DI) water which is environmentally and economically unfriendly, and that the ion doping would add the production cost and complicate the preparation process. In addition, the synthesis of PB needs a lot of chelating agents and auxiliary salts in order to control the co‐precipitation reactions.^[^ ^17^, ^22^, ^23^ ^]^ Without them, the co‐precipitation reactions would become uncontrollable, producing irregular, tiny particles with substantial [Fe(CN)_6_]^4‒^ vacancy and crystal water.^[^ ^23^, ^24^, ^25^, ^26^, ^27^, ^28^ ^]^ A low‐cost, sustainable preparation method is thereby necessary to realize the practical applications of PB.

It seems that the low‐grade water, e.g. sea water, can directly be used to synthesize PB materials. Potential doping ions such as Li^+^, K^+^, Mg^2+^, Ca^2+^, and Al^3+^ can be found in some low‐grade water to realize natural doping, and the use of low‐grade water can achieve goal of low‐cost and sustainable preparation.^[^ ^29^, ^30^, ^31^ ^]^ It is expected that the production cost could be further reduced by recycling and repeated use of the chelating agents and auxiliary salts since their chemical composition and structure are usually kept intact during synthesis.^[^ ^32^, ^33^ ^]^ For the practical applications of PB, it is critical to realize both performance improvement and sustainable preparation.^[^ ^17^, ^22^, ^26^, ^34^, ^35^, ^36^, ^37^ ^]^

Inspired by this, in this work, we propose a promising strategy to use low‐grade water to prepare PB that realizes natural ion doping and low preparation cost. The morphology, structure, composition, and electrochemical performance of the PB materials prepared in different water were systematically studied. The ability of ions intercalation into the PB synthesized in different water was investigated from both thermodynamics and kinetics aspects. The results show that the preparation of PB in sea water can self‐construct Mg^2+^/K^+^ co‐doping in the Na sites that significantly enhances the cycling stability (80.1% capacity retention after 2400 cycles). Theoretical calculation reveals the intrinsic mechanism for structural stabilization of the PB framework by Mg^2+^/K^+^ co‐doping. In addition, we propose a sodium salts (chelating agent and auxiliary salt) recycling method to further address cost‐effective and sustainable preparation of PB.

## 2 Results and Discussion

### 2.1 Preparation and Structural Analyses of the PB Materials

**Figure 1:**
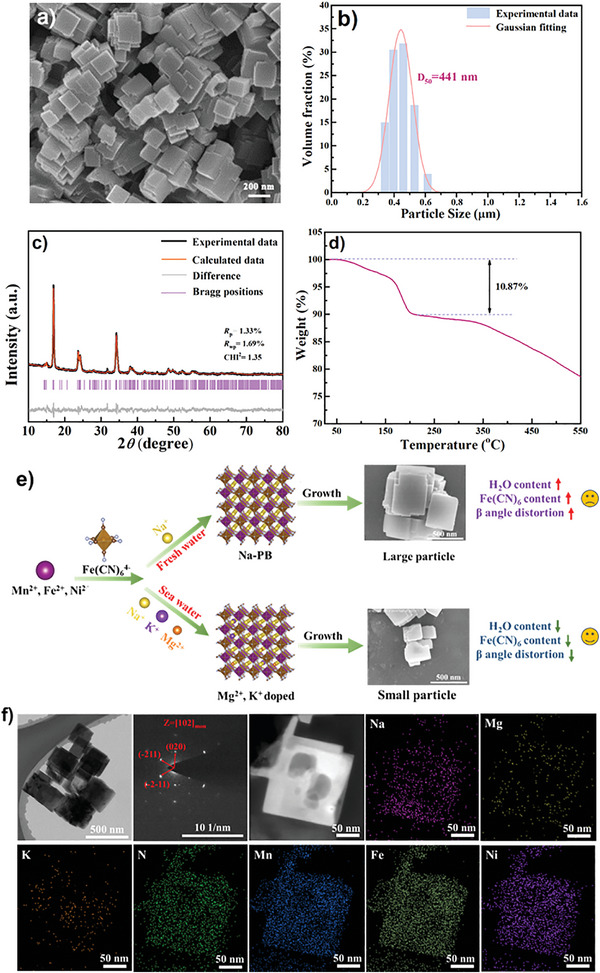
a) SEM image, b) particle size distribution, c) Rietveld refined XRD patterns and d) TG of SW‐PB. e) Schematic illustration of composition, structure and morphology of the PB prepared in different water. f) TEM image, SAED patterns, HAADF‐STEM image and EDX mapping of SW‐PB.

Scanning electron microscopy (SEM) image of the sea water sample (SW‐PB) is shown in **Figure** **1**a. It can be seen that the particles prepared in sea water exhibit an aggregation morphology which is built by several cubic particles with a size of 100‒150 nm. Such a growth mode not only increases the particle size which is favorable for restraining particle dissolution, increasing energy density, and improving electrode processing, but also forms a multi‐edge structure to improve Na‐ion diffusion at the electrode/electrolyte interface. Similar morphology is observed in the other PB samples prepared in DI water (DW‐PB), lake water (LW‐PB), and tap water (TW‐PB) as shown in Figures S1a–S3a (Supporting Information). It seems that the particle size of SW‐PB is smaller than that of DW‐PB, LW‐PB and TW‐PB. The particle size distribution exhibits that the *D*_50_ values of SW‐PB, DW‐PB, LW‐PB, and TW‐PB are 441, 620, 769, and 700 nm, respectively, as shown in Figure 1b, Figures S1b–S3b (Supporting Information). The unique morphology and small size of SW‐PB are beneficial for Na^+^ diffusion and capacity release.

X‐ray diffraction (XRD) Rietveld refinements were performed to investigate the influence of water quality on crystal structure of the PB (Figure 1c; Figures S1c–S3c and Table S1, Supporting Information). Note that the PB materials prepared in different water all exhibit a monoclinic structure (space group: P2_1_/n), suggesting a high Na content in the lattices.^[^ ^38^, ^39^ ^]^ Clearly, DW‐PB, LW‐PB, and TW‐PB show similar crystal parameters while SW‐PB exhibits a reduced crystal volume and a regulated *β* angle (Table S1, Supporting Information). The optimized framework structure and *β* angle can adapt the bond angle changes during phase transition from monoclinic tο cubic and even to tetragonal.^[^ ^22^, ^40^, ^41^ ^]^ Inductively coupled plasma‐atomic emission spectrometry (ICP‐AES) analysis reveals there are trace Mg^2+^ and K^+^ in SW‐PB, but the compositions of LW‐PB and TW‐PB are similar as DW‐PB (Tables S2 and S3, Supporting Information). In order to reveal the effect of Mg^2+^, K^+^ doping on crystal growth, we prepared a series of PB samples in solutions with a gradient Mg^2+^, K^+^ concentration. Obviously, the presence of Mg^2+^, K^+^ does have a structure regulating effect, where the PB exhibits a notable size decrease tendency as Mg^2+^, K^+^ concentration increases (Figures S4 and S5, Supporting Information). Besides, the lattice parameter evolution also demonstrates a structure regulating effect of Mg^2+^, K^+^, which is consistent with the tendency of the sea water synthesis (Figures S6–S8 and Tables S4 and S5, Supporting Information). The above results suggest that Mg^2+^, K^+^ doping indeed has a positive effect on morphology and structure regulation.

Thermogravimetric (TG) results show that the H_2_O content of SW‐PB is 10.87 wt.%, obviously lower than that of DW‐PB (12.04%), LW‐PB (11.90%) and TW‐PB (12.74%) (Figure 1d; Figures S1d–S3d, Supporting Information). The reduced water content in the PB can decrease irreversible side reactions during cycling. To clarify the reason for crystal H_2_O reduction after Mg^2+^, K^+^ introduction, TG test was also performed on the PB samples synthesized in solutions with different Mg^2+^,K^+^ concentrations (Figures S9 and S10, Supporting Information). It can be seen that K+ doping helps to remove crystal H_2_O, while Mg^2+^ doping has an opposite effect. The large ionic size of K^+^ could restrain the occupation of interstitial H_2_O in PB framework as previously reported.^[^ ^13^, ^20^, ^42^, ^43^ ^]^

The difference in composition, structure, and morphology between the PB prepared in fresh water (DI water, lake water and tap water) and sea water is schematically illustrated in Figure 1e. Figure 1f shows the transmission electron microscopy (TEM) image, selected area electron diffraction (SAED) patterns, high‐angle annular dark field (HAADF)‐scanning TEM (STEM) image, and the corresponding energy dispersive X‐ray spectrometry (EDX) mapping. It is evident that uniform Mg^2+^/K^+^ doping is realized in SW‐PB with a monoclinic structure.

**Figure 2:**
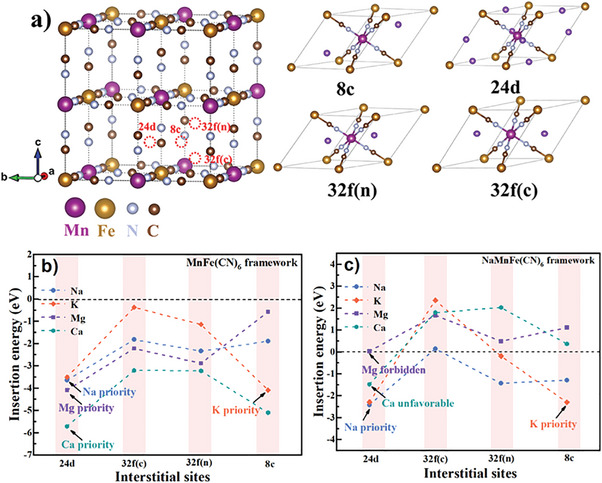
a) Crystal structure of MnFe(CN)_6_ with possible interstitial sites and the corresponding primitive cells with alkali or alkaline earth metal ions occupying at 8c, 24d, 32f(n), and 32f(c) sites. The calculated intercalation energy of Na^+^, Mg^2+^, K^+^, and Ca^2+^ into b) MnFe(CN)_6_ and c) NaMnFe(CN)_6_ framework.

Water quality analysis was performed to clarify the intrinsic mechanism as shown in Table S6 (Supporting Information). Note that alkali or alkaline earth metal ions (Na^+^, K^+^, Mg^2+^, Ca^2+^) constitute the main impurity ions in the low‐grade water, while the contents of transition metal ions such as Mn^2+^, Fe^2+^ and Ni^2+^ are negligible. Therefore, K^+^, Mg^2+^ and Ca^2+^ doping in the Na sites of PB is expected. In order to illustrate the intercalation ability and occupation of Na^+^, Mg^2+^, K^+^ and Ca^2+^ in the PB framework, density functional theory (DFT) calculations were conducted as shown **Figure** **2**. As previously reported,^[^ ^9^, ^34^ ^]^ there are four possible interstitial sites in PB framework with Wyckoff notations of 8c (body center), 24d (face‐center), 32f(c) (displaced from 8c sites toward the C‐coordinated corner), and 32f(n) (displaced from 8c sites toward the N‐coordinated corner) as shown in Figure 2a. The calculated ions insertion energy at different interstitial sites reveals that Na^+^, Mg^2+^, and Ca^2+^ with a small ionic radius of 1.02 Å, 0.72 Å, and 1.00 Å prefer to occupy the 24d sites while the K^+^ with a larger ionic radius of 1.38 Å is more stable at the 8c sites. The insertion energy of the alkali or alkaline earth metal ions into the MnFe(CN)_6_ framework follows the order of *E*_i1_(Ca^2+^, 24d) < *E*_i1_(K^+^, 8c) ≈ *E*_i1_(Mg^2+^, 24d) < *E*_i1_(Na^+^, 24d), suggesting that the intercalation of Ca^2+^, K^+^, and Mg^2+^ into MnFe(CN)_6_ framework is thermodynamically favorable in the Na^+^‐free PB framework (Figure 2b; Table S7, Supporting Information).

Besides, the insertion energy of Mg^2+^, K^+^, and Ca^2+^ into the Na^+^‐rich PB framework was also calculated since the majority of the interstitial sites during practical synthesis would be occupied by Na^+^ as shown in Figure 2c. The insertion energy for Na^+^, Mg^2+^, K^+^ and Ca^2+^ into the NaMnFe(CN)_6_ framework follows the order of *E*_i2_(Na^+^, 24d) ≈ *E*_i2_(K^+^, 24d/8c) < *E*_i2_(Ca^2+^, 24d) << *E*_i2_(Mg^2+^, 24d), suggesting that the intercalation of Na^+^, K^+^ is thermodynamically preferential in the whole process. Of note is that the initial occupied Na^+^ in NaMnFe(CN)_6_ framework is fixed at 24d site because of the lowest thermodynamic energy. Although the intercalation of Ca^2+^ into NaMnFe(CN)_6_ at 24d site is also energetically permitted, its insertion is more difficult than Na^+^ and K^+^ which are energetically more favorable. For Mg^2+^, the intercalation into the NaMnFe(CN)_6_ framework is thermodynamically unfavorable. This indicates that introduction of Mg^2+^ and Ca^2+^ only occurs in the initial stage. The detailed insertion energy for Na^+^, Mg^2+^, K^+^, and Ca^2+^ into MnFe(CN)_6_ and Na_2_MnFe(CN)_6_ framework at different sites is summarized at Tables S7 and S8 (Supporting Information). Besides thermodynamics, the kinetics aspect should also be considered since there is always competition for intercalation between the ions arising from concentration difference. For PB synthesis, the Na^+^ concentration is usually close to its saturation value (over 160 g L^‒1^ in this work) which is much higher than those of the other ions. In contrast, the intercalation of Ca^2+^ is kinetically shielded because of its rather low concentration (315.55 mg L^‒1^) in sea water. The low concentration of the impurity ions in lake water and tap water also makes their insertion kinetically impossible. But in sea water, the shielding effect of Na^+^ is greatly weakened since the K^+^ and Mg^2+^ have a relatively higher concentration of 582.89 and 1001.29 mg L^‒1^, respectively, so as to realize K^+^ and Mg^2+^ intercalation. As a result, a self‐construction of K^+^/Mg^2+^ co‐doping in the PB was achieved by synthesizing it in sea water.

### 2.2 Electrochemical Performance and Mechanism of the PB Materials

**Figure 3:**
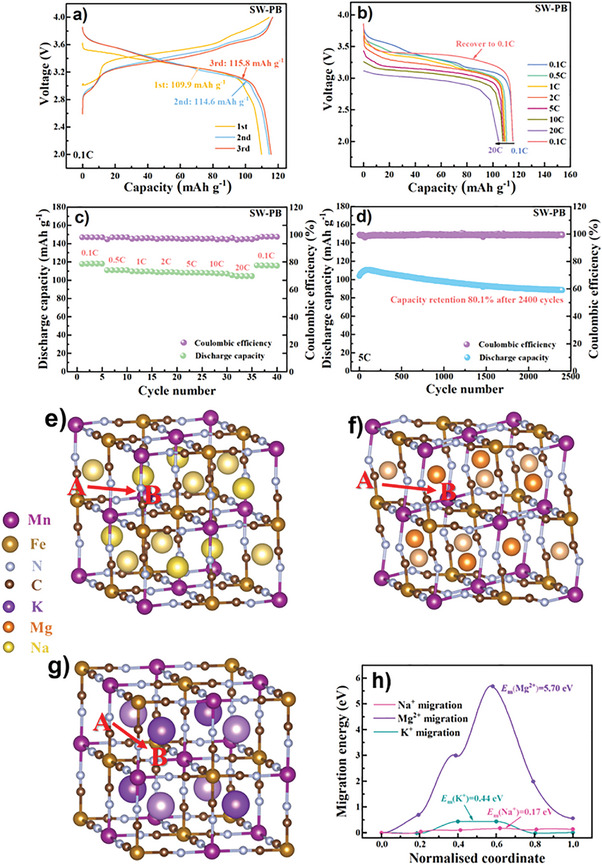
Electrochemical performance of SW‐PB. a) Voltage curves in the initial three cycles at 0.1 C. b) Discharge curves and c) discharge capacity at various current densities from 0.1 C to 20 C, and d) cycling stability at 5 C. The migration models for e) Na^+^, f) Mg^2+^, and g) K^+^ to move from one interstitial site ([A] site) to adjacent interstitial site ([B] site) and h) the corresponding migration energy barriers for Na^+^, Mg^2+^, and K^+^ to move from A site to B site.

Electrochemical performance of SW‐PB, DW‐PB, TW‐PB, and LW‐PB was investigated by galvanostatic cycling in a voltage range of 2.0–4.0 V as shown in **Figure** **3** and Figures S11‒S13 (Supporting Information). It can be seen that SW‐PB delivers a comparable discharge capacity of 115.8 mAh g^‒1^ at 0.1 C as DW‐PB, TW‐PB, and LW‐PB (121.5, 118.5, and 118.5 mAh g^‒1^, respectively). Based on the chemical formula of SW‐PB, the theory discharge capacity contributed by Mg^2+^ and K^+^ is 4.8 mAh g^‒1^,^[^ ^43^ ^]^ which is close to the decreased value compared with DW‐PB (Figure 3a; Figure S11a, Supporting Information), indicating that Mg^2+^ and K^+^ may stay in SW‐PB lattice during charge. In order to prove this assumption, elemental analysis of Na^+^, K^+^, and Mg^+^ in SW‐PB at pristine and charge state was carried out (Table S9, Supporting Information). Note that the Mg^2+^ and K^+^ can well stay in the interstitial sites upon charge to support structural stability of the PB and the capacity comes mainly from extraction/insertion of Na^+^. The residual Na^+^ at charge state is due to the doping of inert Ni^2+^.^[^ ^17^, ^44^, ^45^ ^]^ Although there is possible blocking effect of Mg^2+^and K^+^ for Na^+^ diffusion, SW‐PB exhibits superior rate performance with 90.4% capacity retained at 20 C relative to that at 0.1 C.

Cycling performance of the four PB samples is compared as seen in Figure 3d and Figures S11d–S13d (Supporting Information). Note that SW‐PB with Mg^2+^/K^+^ co‐doping exhibits the best cycling stability with a 93.9% capacity retention after 500 cycles at 5 C, superior to DW‐PB (88.6%), LW‐PB (88.4%) and TW‐PB (87.6%). Even after 2400 cycles at 5 C, SW‐PB can still maintain a high capacity retention of 80.1%. The significantly enhanced cycling stability for SW‐PB indicates that the introduced Mg^2+^ and K^+^ in the PB can act as “pillar” to well stabilize the structure during repeated cycling. In order to further illustrate the “pillar” effect of Mg^2+^ and K^+^ in SW‐PB, the migration energy barriers (*E*_m_) of Na^+^, K^+^, and Mg^2+^ in PB framework (Figure 3e‒h) was calculated through DFT.^[^ ^46^ ^]^ The migration energy required for Na^+^, Mg^2+^, and K^+^ to move from A site to B site in PB framework is 0.17 eV, 5.70 eV, and 0.44 eV, respectively. Clearly, the migration energy barriers of Mg^2+^ and K^+^ (especially for Mg^2+^) in the PB is obviously larger than that of Na^+^ because of the higher charge density of Mg^2+^ and larger ionic size of K^+^. The immovability of K^+^ and Mg^2+^ upon cycling makes them act as the “pillar” to well support the PB framework, thus significantly enhancing cycling performance.

In order to reveal the impacts of impurities such as Al^3+^, Ca^2+^ and organic substances on electrochemical performance of SW‐PB, we also tested the electrochemical performance of the PB directly synthesized in solution with comparable Mg^2+^ K^+^ concentration as that in sea water (Figure S14, Supporting Information). It can be seen that the as‐prepared PB exhibits a comparable rate capability and cycling stability as SW‐PB, suggesting that the impurities in sea water have limited effects on PB performance. In addition. we investigate the electrochemical performance of SW‐PB washed by sea water to further show the influence of the impurities (Figure S15, Supporting Information). Note that the impurities in sea water would cause a negative impact on electrochemical performance with somewhat degraded cycle life and fluctuated coulombic efficiency, indicating an aggravated side reaction caused by the impurities. The above results suggest that the impurities in natural water indeed have adverse effects on electrochemical performance. But after DI water washing of the product, the influence of the impurities can be minimized. The electrochemical performance comparison of the above PB samples is summarized in Table S10 (Supporting Information).

Based on the Mg^2+^,K^+^ concentration in sea water, we further compare the electrochemical performance of PB samples synthesized in solutions with different Mg^2+^,K^+^ concentrations to obtain a proper Mg^2+^, K^+^ doping amount as shown in Figures S16‒S19 (Supporting Information). The ionic concentration, materials composition and electrochemical performance are summarized in Tables S11 and S12 (Supporting Information). Of note is that the level of Mg^2+^, K^+^ doping in Na site is closely related to Mg^2+^,K^+^ concentration in the precursors. All the Mg^2+^, K^+^ doped samples show enhanced cycling stability compared to DW‐PB, suggesting that Mg^2+^,K^+^ co‐doping indeed has a positive effect on framework stability but the degree of the enhancement effect varies with the doping level. The improvement degree for sample 1 with less or sample 4 with excessive Mg^2+^, K^+^ addition is lower than that of sample 2 and sample 3 with moderate Mg^2+^,K^+^ addition. Besides, a decrease in discharge capacity as well as rate capability is observed with excessive Mg^2+^, K^+^ addition. In a word, a strategy of moderate Mg^2+^, K^+^ doping can both enhance cycling stability and keep a high discharge capacity. Exactly, the Mg^2+^, K^+^ concentration in sea water is appropriate for moderate Mg^2+^, K^+^ doping, suggesting that the sea water can act as a reaction medium to achieve natural yet appropriate Mg^2+^, K^+^ doping in PB with improved electrochemical performance.

**Figure 4:**
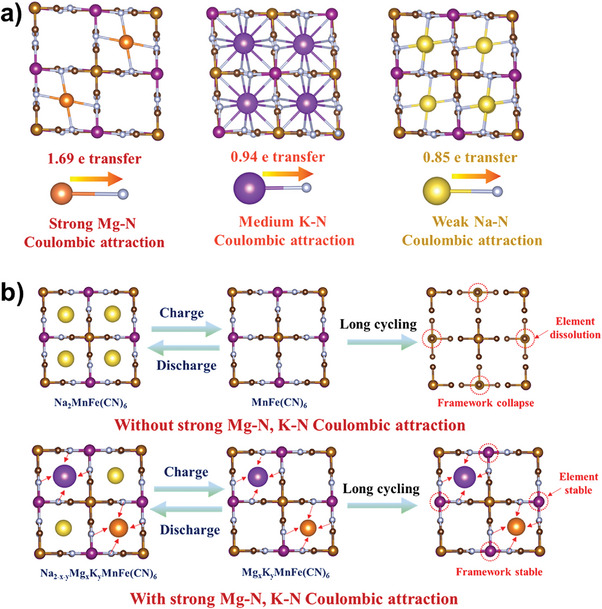
a) Optimized structures of Na‐PB, Mg‐PB, and K‐PB and calculated charge transfer values from Na, Mg, and K to N atoms. b) Stabilizing mechanism of the PB framework by Mg^2+^/K^+^ co‐doping.

In order to understand the “pillar” effect of Mg^2+^ and K^+^ ions in PB, we investigated the electronic structure of the Na‐PB, Mg^−^PB, and K‐PB through DFT calculations as shown in **Figure** **4**a and Figure S20 (Supporting Information). The average transferred charges from Na to N, Mg to N, and K to N atom in Na‐PB, Mg‐PB, and K‐PB framework are 0.85e, 1.69e, and 0.94e, respectively (Table S13, Supporting Information). The increased charge transfer between Mg (or K) and N atoms indicates a stronger Coulombic attraction of Mg‒N and K‒N than Na‒N. The strongly bonded N atoms by Mg and K can well restrain the structural collapse after repetitive Na extraction/insertion which is well proved by the inhibited dissolution of the transition metal elements, where the Mn/Ni content of SW‐PB after 300 cycles is obviously higher than that of DW‐PB (Table S14, Supporting Information). Furthermore, we calculated the Bader charges variation on N atoms for Na‐PB, Mg^2+^ doped Na‐PB and K^+^ doped Na‐PB during Na insertion/extraction process in order to well explain the framework stabilization effects of Mg^2+^,K^+^ doping. It can be seen that the existed Mg‒N, K‒N Coulombic interaction in PB framework can well reduce the charge density variation of the neighboring N atoms during Na^+^ extraction/insertion process, enabling a restrained Jahn‐Teller effect of the central Mn atom (Tables S15‒S17, Supporting Information).^[^ ^47^ ^]^ The well‐maintained framework structure after Mg^2+^,K^+^ doping during Na^+^ insertion/extraction process is also proved by charge density difference as shown in Figure S21 (Supporting Information). Clearly, there is negligible bonding variation of Mn‒N, N‒C, and C‒Fe during Na extraction/insertion process for Mg^2+^ doped Na‐PB and K^+^ doped Na‐PB, further demonstrating a better structural stability. The stabilizing mechanism of the PB framework by Mg^2+^/K^+^ co‐doping is schematically presented in Figure 4b.

**Figure 5:**
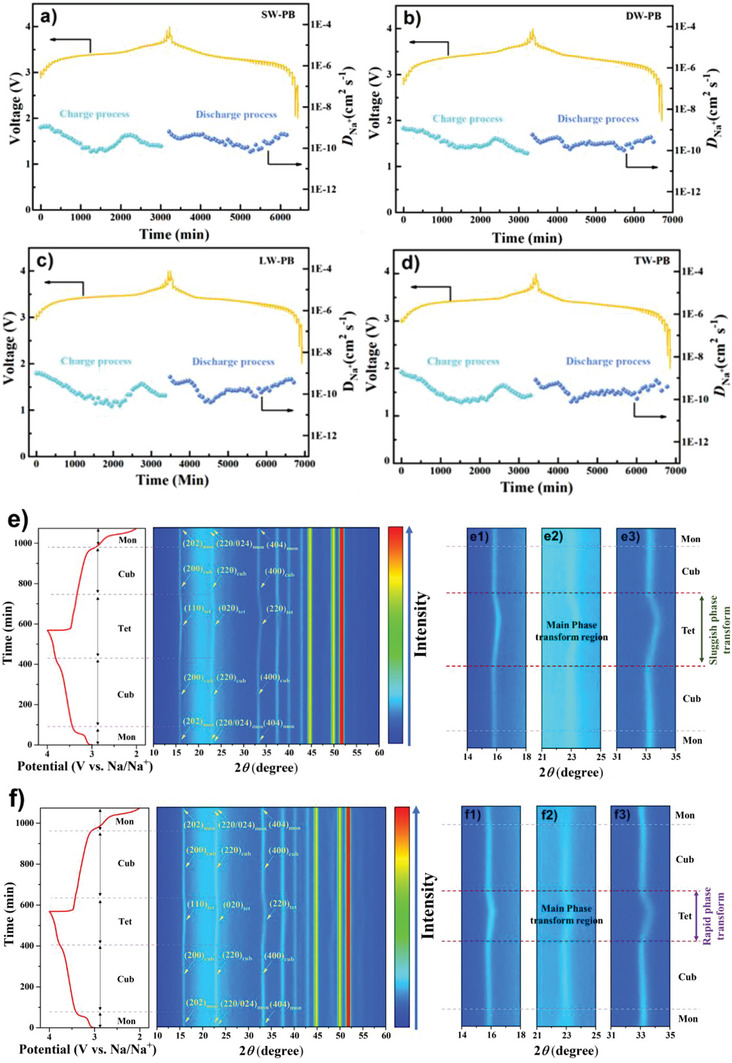
GITT curves and the *D*_Na_^+^ values of a) SW‐PB, b) DW‐PB, c) LW‐PB and d) TW‐PB. 2D contour plots of in‐situ XRD patterns of e) SW‐PB and f) DW‐PB during the initial charge/discharge process.

The impact of Mg^2+^/K^+^ co‐doping on Na^+^ diffusion and phase transition was investigated by galvanostatic intermittent titration technique (GITT) and in‐situ XRD as shown in **Figure** **5**. The average diffusion coefficients (*D*_Na_^+^) values for DW‐PB, SW‐PB, LW‐PB, and TW‐PB at discharge process are 2.34×10^‒10^, 2.33×10^‒10^, 1.64×10^‒10^, 2.56×10^‒10^ cm^2^ S^‒1^, respectively. It is evident that the *D*_Na_^+^ values are comparable, indicating that the trace Mg^2+^ and K^+^ in the PB framework would not block the bulk Na^+^ diffusion. Furthermore, the smaller particle size of SW‐PB with Mg^2+^/K^+^ doping is beneficial for Na^+^ diffusion. In‐situ XRD suggests that SW‐PB exhibits the similar structural evolution as DW‐PB with reversible monoclinic‐cubic‐tetrahedral (Mon‐Cub‐Tet) and Tet‐Cub‐Mon phase transitions during Na^+^ insertion/extraction process (Figure 4e,f). As for the phase transition rate, it is clear that SW‐PB has a slow structural variation from Cub to Tet phase than DW‐PB (Figure 5e1‒e3,f1‒f3). Notice that the Cub‐Tet transform accounts for the main volume variation for the PB and the sluggish phase transform after Mg^2+^/K^+^ co‐doping can relieve the structural changes which is good for enhanced cycling stability. To further understand the structure stabilizing effect of Mg^2+^,K^+^ doping, the structural variation of SW‐PB during charge‐discharge process is investigated through XRD Rietveld refinements (Figure S22, Supporting Information) and the corresponding crystal parameters are summarized in Table S18 (Supporting Information). The results indicate that the crystal volume variation for SW‐PB is only 0.17% after charge, which is far less than that of DW‐PB (0.60%), suggesting a superior structure stabilizing effect of Mg^2+^,K^+^ co‐doping. The electrochemically inert Mg^2+^,K^+^ ions can act as “pillar” to well restrain phase transition and enhance structural stability as previously reported.^[^ ^48^, ^49^ ^]^ In a word, Mg^2^/K^+^ co‐doping in the PB can slow down structural changes during repetitive Na^+^ insertion/extraction because of the strong K‒N and Mg‒N interactions with stabilized crystal lattices.

In order to understand the role of Mg^2+^/K^+^ co‐doping in maintaining the structural stability of the PB framework during cycling, the morphology evolution was checked after cycling as shown in Figure S23 (Supporting Information). Note that the majority of SW‐PB particles can well maintain the initial cubic shape after 50, 150, and 300 cycles without severe particle dissolution and breach. By contrast, the particles of DW‐PB, LW‐PB, and TW‐PB gradually become irregular with a rough surface after prolonged cycling, indicating a severe particle corrosion (Figures S24‒S26, Supporting Information). The morphology variation further demonstrates that the Mg^2+^/K^+^ co‐doping endows the PB with a more rigid structure which can well tolerate repeated Na^+^ extraction/insertion and restrain the particle dissolution. It seems that the sea water can act as water source to synthesize high‐performance PB by self‐construction of Mg^2+^/K^+^ co‐doping. Importantly, the strategy of direct use of sea water well fits the goal of large‐scale, low ‐cost PB preparation and sustainable development.

### 2.3 Sustainable Preparation of PB using Recycled Salts

Considering the fact that the waste water after PB preparation contains substantial sodium salts (sodium citrate, Na_2_SO_4_), an attempt was made to directly use the waste water to prepare PB. The waste water was collected after the preparation of DW‐PB as shown in Figure S27 (Supporting Information). The morphology, structure, and electrochemical performance of the PB prepared directly using waste water were compared with those of DW‐PB. The results indicate that although the majority of the PB particles prepared with the waste water exhibit a large, regular cube morphology, there are some tiny and irregular particles, indicating an uncontrolled co‐precipitation process (Figure S28a, Supporting Information). The particle size distribution also shows an obviously reduced *D*_50_ (423 nm), suggesting a quick co‐precipitation reaction in the waste water (Figure S28b, Supporting Information). TG and XRD analyses show that the waste water sample still maintain a monoclinic structure but the crystal water content (12.54%) is increased compared to DW‐PB (Figure S28c,d, Supporting Information). The chemical formula of the waste water sample is Na_1.61_Mn_0.57_Fe_0.20_Ni_0.23_[Fe(CN)_6_]_0.90_·2.26H_2_O, which has an increased Fe(CN)_6_ defect (Tables S19 and S20, Supporting Information). Water quality analysis shows that there are a large number of Mn, Fe, and Ni ions in the waste water (mainly Fe(CN)_6_ and Ni ions), which would affect crystallization of the PB (Table S21, Supporting Information).

The waste water sample also shows a degraded electrochemical performance as shown in Figure S29 (Supporting Information). The sample exhibits a reduced discharge capacity of 115.5 mAh g^‒1^ at 0.1 C and deteriorated capacity retention of 88.7% at 20 C relative to that at 0.1 C (Figure S29a‒c, Supporting Information). Furthermore, when current density is back to 0.1 C, the discharge capacity cannot be well recovered, indicating a structural destruction after high‐rate cycling. The sample also shows a relatively poor cycling stability with 82.8% retention after 500 cycles at 5 C (Figure S29d, Supporting Information). As a result, direct use of the waste water for PB synthesis is unfavorable and purification of the waste water is necessary.

It should be noted that the composition of the waste water is rather complex containing sodium citrate, Na_2_SO_4,_ and other residual ions such as Fe(CN)_6_^4‒^, Mn^2+^, Fe^3+^, and Ni^2+^ (Figure S30a and Table S21, Supporting Information). Here, a simple purifying route is proposed by using precipitation and ion exchange route to remove the impurity ions in the waste water. The sodium citrate and Na_2_SO_4_ were separated based on their solubility difference. XRD test reveals that the recycled sodium citrate and Na_2_SO_4_ exhibit the almost same patterns as the pristine ones (Figure S30b,c, Supporting Information). The XRD results and the calculated recycling rate indicate that the proposed purifying method is highly efficient. ICP‐AES results also demonstrate a high purity of the recycled salts solution (Table S21, Supporting Information). The detailed process of the waste water purification and salts separation is shown in the Experimental Section and Figure S31 (Supporting Information). Clearly, the morphology, structure, particle size, TG and chemical formulas of the PB using the recycled salts are comparable with those of DW‐PB (Figure S32 and Tables S19–S22, Supporting Information).

**Figure 6:**
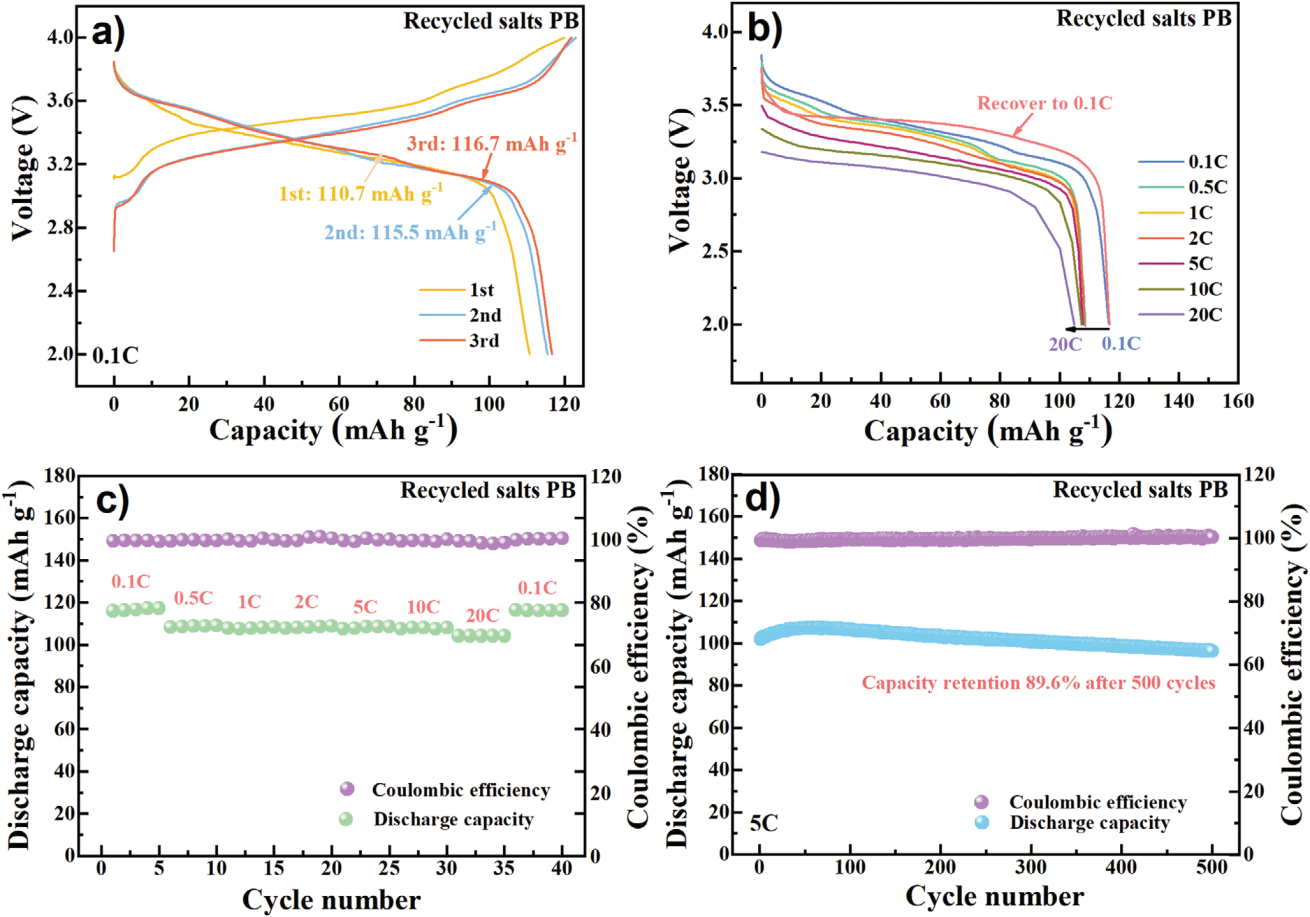
Electrochemical performance of the recycled salts sample. a) Voltage curves of the initial three cycles at 0.1 C. b) Discharge curves and c) discharge capacity at various current densities from 0.1 to 20 C, and d) cycling stability at 5 C.

Electrochemical performance of the recycled salts PB sample is presented in **Figure** **6**. The initial capacity (116.7 mAh g^−1^ at 0.1 C), rate capability (89.6% at 20 C relative to that at 0.1 C) and cycle life (89.6% after 500 cycles at 5 C) of the PB are comparable with those of DW‐PB (Table S23, Supporting Information). The results suggest that the proposed recycling route is feasible and efficient. It also suggests trace amounts of impurity ions (< 10 mg L^‒1^) can hardly affect the crystallization of the PB because of the kinetically shielding effect of the high‐concentration precursor salts. Note that sodium citrate and Na_2_SO_4_ are the main costs for PB synthesis and the sodium salts recycling strategy would obviously reduce average raw materials cost (Tables S24 and S25, Supporting Information). For example, the reuse of sodium citrate and the Na_2_SO_4_ for 9 times (total 10 times) can reduce the average raw materials cost by ≈40%.

**Figure 7:**
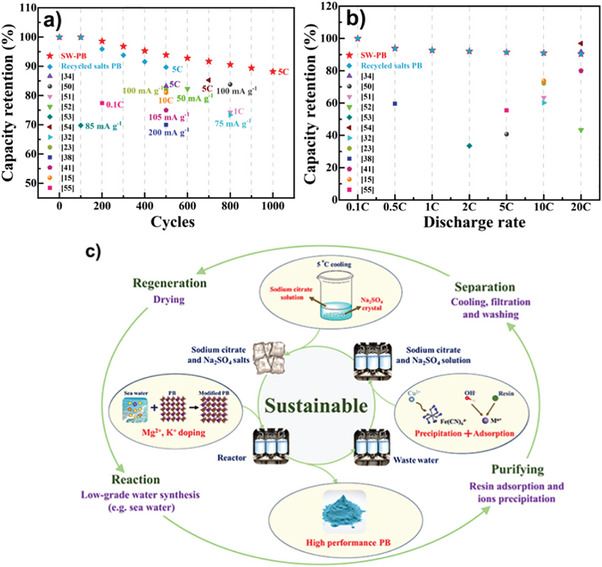
a) Cycling performance and b) rate performance comparison of SW‐PB and the recycled salts sample with other Mn‐based PB materials. c) Schematic illustration of sustainable synthesis of high‐performance PB materials.

To highlight superior performance of our PB samples, we compare the electrochemical performance of SW‐PB and the recycled salts sample with those of the previously reported Mn‐based PB materials (**Figure** **7**a,b).^[^ ^15^, ^23^, ^32^, ^34^, ^38^, ^41^, ^50^, ^51^, ^52^, ^53^, ^54^, ^55^ ^]^ Clearly, the cycling stability and rate capability of SW‐PB and the recycled salts sample are better than those of the other Mn‐based PB materials, demonstrating feasibility of using sea water and recycled salts in PB synthesis. The self‐constructed Mg^2+^/K^+^ co‐doping in Na sites can improve cycling stability because of the stronger Mg‒N and K‒N Coulombic attraction which can restrain the structure collapse and elements dissolution during repetitive Na^+^ insertion/extraction. This work suggests that high‐quality PB can be obtained by a cost‐effective and sustainable route (Figure 7c).

## 3 Conclusions

A self‐constructed trace Mg^2+^/K^+^ co‐doped PB was synthesized in sea water which exhibits a superior rate performance (90.4% at 20 C relative to that at 0.1 C) and extremely long cycle life (80.1% retention after 2400 cycles at 5 C). The insertion of Mg^2+^ and K^+^ into PB is both thermodynamically and kinetically permitted during the sea water synthesis. DFT calculation reveals the “pillar” effect Mg^2+^ and K^+^ which is essential to stabilize the PB framework. The enhanced Coulombic attraction of Mg‒N and K‒N pairs can relieve the phase transition and restrain the elements dissolution during repetitive Na^+^ insertion/extraction. In addition, a feasible purification and separation strategy was proposed to recycle the sodium salts, so as to prepare PB in a cost‐effective and sustainable route. This work proves that high performance and low production cost of PB can be simultaneously achieved which is critical for its practical applications in large‐scale energy storage.

## 4 Experimental Section

### Preparation of PB Materials

The PB materials were synthesized via a chelating agent‐assisted coprecipitation route.^[^ ^56^, ^57^ ^]^ For a typical synthesis in DI water, 4.8 mmol of MnSO_4_ **·**H_2_O, 1 mmol of FeSO_4_ **·**7H_2_O, 0.4 mmol NiSO_4_ **·**6H_2_O, 0.02 mol Na_3_C_6_H_5_O_7_ **·**2H_2_O and 22.5 g Na_2_SO_4_ were dissolved in 50 mL of DI water to form solution A. 1.2 mmol of MnSO_4_ **·**H_2_O, 1 mmol of FeSO_4_ **·**7H_2_O, 1.6 mmol NiSO_4_ **·**6H_2_O, 0.02 mol Na_3_C_6_H_5_O_7_ **·**2H_2_O and 5 g Na_2_SO_4_ were dissolved in 50 mL of DI water to form solution B. 10 mmol of Na_4_[Fe(CN)_6_]**·**10H_2_O was dissolved in 100 mL of DI water to form solution C. Both solution A and B were bubbled with N_2_ flow to avoid oxidation of divalent iron ions. Then, solution B and C were added dropwise into solution A simultaneously through a peristaltic pump under magnetic stirring at 60 °C in an oil bath. The feeding speed of solution B and C was 0.5 mL min^‒1^ and 1 mL min^‒1^, respectively. After the reaction, the white suspension was aged for 3 h under stirring with N_2_ flow at 60 °C. The white precipitate was separated and washed by centrifugation in DI water and absolute alcohol. Finally, the PB material was obtained after drying at 110 °C for 24 h in vacuum (labeled as DW‐PB). Similarly, lake water, sea water and tap water instead of DI water were used to synthesize PB materials with a same process which were labeled as LW‐PB, SW‐PB and TW‐PB, respectively. The lake water is from Qiandao Lake (Hangzhou), the sea water is from Yintan Beach (Weihai), and the tap water is from Zhejiang University (Hangzhou). All of the other chemical reagents for synthesis were purchased from Sinopharm Chemical Reagent Co., Ltd (analytical reagents). The synthesis method for PB samples with different Mg^2+^, K^+^ contents is similar to that for SW‐PB except that the sea water was replaced by DI water with a desirable amount of MgCl_2_ and KCl.

### Na Salts Recycling and Reuse

The waste water was first concentrated to 110 mL in an oil bath at 95 °C followed by vacuum suction filtration. The preparation process of the PB material using the waste water is the same as DW‐PB except that DI water in solution A and B was replaced by the waste water (57 mL in solution A and 53 mL in solution B) without using Na_3_C_6_H_5_O_7_·2H_2_O and Na_2_SO_4_. The preparation of solution C is the same as DW‐PB by using DI water as the solvent. After the reaction, the PB was separated and washed by centrifugation in DI water and absolute alcohol followed by drying at 110 °C for 24 h in vacuum. The separation of Na_3_C_6_H_5_O_7_·2H_2_O and Na_2_SO_4_ from the waste water adopts a simple solubility difference strategy and the impurity ions were removed through precipitation processes assisted by ion exchange using an ion‐exchange resin (Purolite, S930Plus). The detained separation process is presented in Figure S31 (Supporting Information). Specifically, 0.01 mol CuSO_4_ **·**5H_2_O was first added into 100 mL of the waste water with heating at 95 °C for 2 h to remove the residual Fe(CN)_6_^4‒^. Then, the suspension was suction filtered and 0.03 mol NaOH was added into the filtrate to remove the Mn^2+^, Fe^3+^, Ni^2+^, and Cu^2+^. After sufficient reaction at 95 °C and suction filtration, 0.1 mol L^‒1^ H_2_SO_4_ was added into the filtrate dropwise to adjust the solution pH. The obtained solution was further purified by using ion‐exchange resin to adsorb the remaining ions (mainly Cu^2+^) in a chromatography column. After that, the solution was directly concentrated to 15 mL at 95 °C and cooled to 5 °C to crystallize Na_2_SO_4_ followed by suction filtration. Finally, the filtrate was heated at 95 °C with water evaporation to obtain Na_3_C_6_H_5_O_7_·2H_2_O. The obtained Na_2_SO_4_ and Na_3_C_6_H_5_O_7_ were washed with 95% alcohol solution to further remove the impurity ions and the drying was conducted in a vacuum oven at 110 °C. The preparation of the recycled salts sample is the same as DW‐PB by using recycled Na_2_SO_4_ and Na_3_C_6_H_5_O_7_·2H_2_O.

### Material Characterization

Crystal structure of the PB materials was characterized by XRD on a powder diffractometer (Rigaku D/Max‐2550 pc) with Cu Kα radiation (*λ* = 1.541 Å). TG tests were performed in N_2_ atmosphere on a Netzsch LFA467 device. Chemical composition of the PB materials and ion concentration of the various water sources were determined using ICP‐AES (Agilent 720ES system). Morphology of the samples was characterized by field‐emission SEM (Hitachi SU8010 microscope). Size distribution of the PB materials was determined by Zeta potential analysis (Malvern Zetasizer Nano ZS90). The microstructure of the PB was analyzed by TEM on a Tchnai F20 microscope.

### In‐Situ XRD Measurement

In‐situ XRD tests were conducted on a Rigaku MiniFlex 600. To increase the peak intensity of the XRD patterns and eliminate peaks of the current collector, a free standing electrode was made by pressing active material and carbon nanotube (CNT) into a pellet at mass ratio of 7:3 under 4 MPa. The cathode pallet was placed in contact with Beryllium window followed by adding separator (glass fiber), electrolyte (1 M NaPF_6_ in propylene carbonate (PC): fluoroethylene (FEC), 9:1 in volume), and sodium metal anode in sequence. The fabrication of the in‐situ cell was conducted in an Ar‐filled glove box. During in‐situ XRD measurement, the cell was charged and discharged at 0.1 C (1 C = 150 mA g^−1^) and the patterns were collected every 15 min to capture the structural evolution of the cathode materials.

### Electrochemical Measurements

CR2025‐type coin cells were assembled in an argon‐filled glovebox, using Na metal disk as anode, glass fiber (GF/D, Whatman) as separator, and an electrolyte containing 1 M NaPF_6_ in ethyl methyl carbonate (EMC) and PC (1:1 in volume) with 4 wt.% FEC additive (DoDoChem). For the preparation of cathode, 70 wt.% active material, 10 wt.% polyvinylidene fluoride (PVDF), and 20% carbon nanotubes (CNT) were mixed in N‐methyl pyrrolidone (NMP) to form a slurry. The slurry was pasted on Al foil followed by drying under vacuum at 110 °C for 24 h. Charge and discharge cycling of the coin cells was conducted on a battery testing system (Neware Technology Ltd, China) at room temperature over a voltage range of 2–4 V (versus Na/Na^+^). GITT was used to measure the Na^+^ chemical diffusion coefficients using a current pulse of 15 mA g^‒1^ for 10 min and relaxation time for 1 h until a desired cut‐off voltage was reached. Before the GITT measurements, the cells were charged and discharged at 15 mA g^‒1^ for three cycles. Na^+^ diffusion coefficients were calculated by the following equation^[^ ^58^, ^59^ ^]^:

(1) $$\;D_{Na^{+}}=\frac{4}{\pi \tau }\;{\left(\frac{m_{B}V_{M}}{M_{B}S}\right)}^{2}{\left(\frac{\Delta E_{S}}{\Delta E_{\tau }}\right)}^{2}$$

where τ represents the pulsing time, *m*_B_ is the mass of active materials, *M*_B_ and *V*_M_ are the molar mass and molar volume, respectively, *S* is the surface area of the electrode, Δ*E*_s_ is the potential change during a steady pulse, and Δ*E*_τ_ is the potential change during a constant current pulse.

### Computational Methods

All the calculations were performed by applying the spin‐polarized DFT method using the Vienna ab initio simulation package (VASP) with the projector‐augmented wave (PAW) method.^[^ ^60^, ^61^ ^]^ The generalized gradient approximation (GGA) with Perdew‐Burke‐and Ernzerhof (PBE) was used for the exchange‐correlation functional. In order to simplify the calculation model, the higher symmetry Mn‐PB framework was adopted to replace the Ni,Fe doped Mn‐PB framework. The Hubbard U values (*U*_eff_) of 5.0 eV for Mn and 3.0 eV for Fe were set to describe the d‐part of the Hamiltonian based on the previous literature.^[^ ^62^ ^]^ The plane‐wave energy cutoff was set above 400 eV. For structure optimization, the ions force convergence and the electronic energy convergence criteria were set to 0.05 eV Å^‒1^ and 10^−5^ eV, respectively. A 3×3×3 Monkhorst‐Pack k‐point mesh was used for the Brillouin zones.

The reversible insertion reaction of the alkali cations A (A represents Na^+^, K^+^, Mg^2+^, and Ca^2+^) into MnFe(CN)_6_ and relative insertion energy can be written as follows:

(2) $$\begin{array}{ccc} \mathrm{MnFe}{\left(\mathrm{CN}\right)}_{6}+A\to \mathrm{AMnFe}{\left(\mathrm{CN}\right)}_{6},E_{i1} & = & E\left[\mathrm{AMnFe}\left(\mathrm{CN}\right)\right]\\ & & -E\left[\mathrm{MnFe}{\left(\mathrm{CN}\right)}_{6}\right]-E\left[A\right] \end{array}$$

The reversible insertion reactions of the alkali cations A into NaMnFe(CN)_6_ and relative insertion energy are as follows:

(3) $$\begin{array}{ccc} \mathrm{NaMnFe}{\left(\mathrm{CN}\right)}_{6} & + & A\to \mathrm{ANaMnFe}{\left(\mathrm{CN}\right)}_{6},E_{i2}=E\left[\mathrm{ANaMnFe}\left(\mathrm{CN}\right)\right]\\ & - & E\left[\mathrm{NaMnFe}{\left(\mathrm{CN}\right)}_{6}\right]-E\left[A\right] \end{array}$$

where *E*[AMnFe(CN)_6_], *E*[NaMnFe(CN)_6_], *E*[ANaMnFe(CN)_6_], *E*[MnFe(CN)_6_] and *E*[A] represent total energy of AMnFe(CN)_6_, NaMnFe(CM)_6_, ANaMnFe(CN)_6_, MnFe(CN)_6_ and metallic A calculated using DFT. The calculated energy of Na, Mg, K, and Ca atom are ‒1.30 eV, ‒1.38 eV, ‒1.02 eV, and ‒1.92 eV, respectively. The framework structure is fully optimized before use.^[^ ^63^ ^]^

The minimum energy path way search of ion migration was conducted with the climbing image nudged elastic band (CI‐NEB) method. The ions force convergence and the electronic energy convergence criteria were set to 0.05 eV Å^‒1^ and 10^−7^ eV.^[^ ^21^, ^64^ ^]^

The electronic properties calculations were conducted on a fully geometry‐optimized Na_2_MnFe(CN)_6_ (Na‐PB), K_2_MnFe(CN)_6_ (K‐PB) and MgMnFe(CN)_6_ (Mg‐PB) cells. The occupation sites of Na, K, and Mg atoms were based on the above calculation results of *E*_i1_.

## Conflict of Interest

The authors declare no conflict of interest.

## Supporting information



Supporting Information

## Data Availability

The data that support the findings of this study are available from the corresponding author upon reasonable request.
