# Supplementary material for: A Self‐Constructed Mg2+/K+ Co‐Doped Prussian Blue with Superior Cycling Stability Enabled by Enhanced Coulombic Attraction
Source: Adv Sci (Weinh). 2024 Sep 20;11(42):2406842. doi: 10.1002/advs.202406842 (PMC11558122; doi:10.1002/advs.202406842)
Supplement: Supplementary file 1 — Supporting Information [file ADVS-11-2406842-s001.docx]

Supporting Information

**A Self-Constructed Mg^2+^/K^+^ Co-Doped Prussian Blue with Superior Cycling Stability Enabled by Enhanced Coulombic Attraction**

Zheng Xu,^a^ Fengqin Chen,^b^ Yinda Li,^c^ Yunhao Lu,^c^ Aijun Zhou,^d^ Jicheng Jiang,^d^ Xiongwen Xu,^e^ Jian Tu,^e^ Bin Pan,^f^ Fang Chen,^g^ Yi Huang,^a^ Xinbing Zhao,^a^ and Jian Xie^a,^^[[1]](#footnote-1)^*

^a^ State Key Laboratory of Silicon and Advanced Semiconductor Materials, School of Materials Science and Engineering, Zhejiang University, Hangzhou 310058, China

^b^ Shaanxi Coal Chemical Industry Technology Research Institute, Xi’an 710100, China

^c^ School of Physics, Zhejiang University, Hangzhou 310058, China

^d^ Yangtze Delta Region Institute (Huzhou), University of Electronic Science and Technology of China, Huzhou 313000, China

^e^ LI-FUN Technology Corporation Limited, Zhuzhou 412000, China

^f^ Yuna Technology Corporation Limited, Hangzhou 311121, China

^g^ Department of Chemistry, Zhejiang University, Hangzhou 310058, China


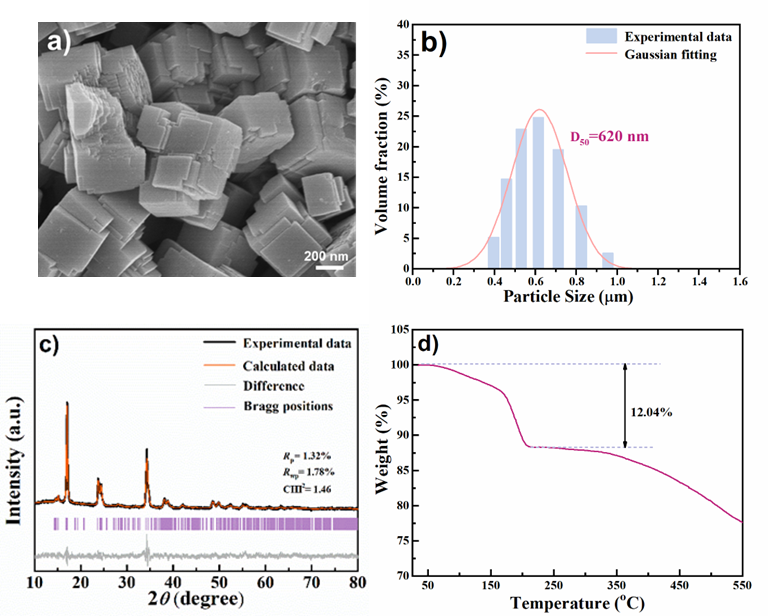


**Figure S1.** a) Morphology, b) particle size distribution, c) Rietveld refined XRD patterns and d) TG of DW-PB.


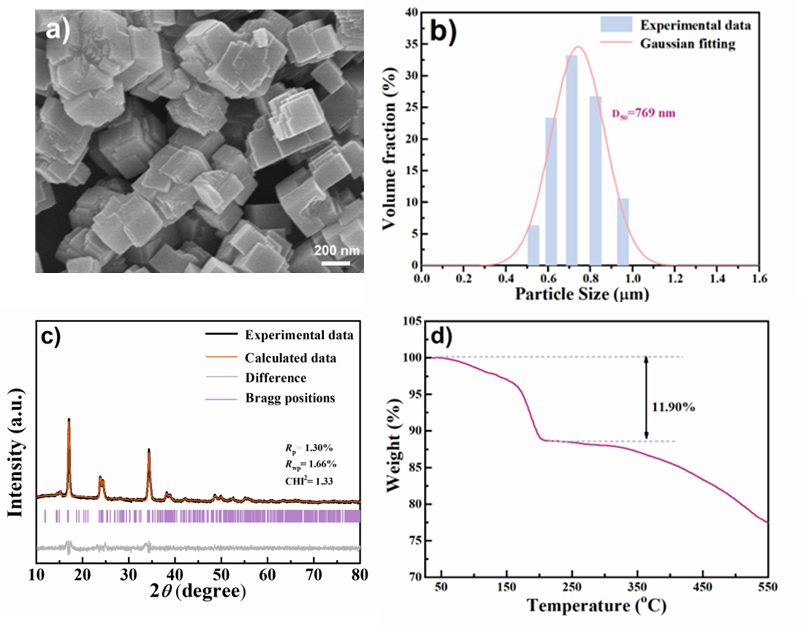


**Figure S2.** a) Morphology, b) particle size distribution, c) Rietveld refined XRD patterns and d) TG of LW-PB.


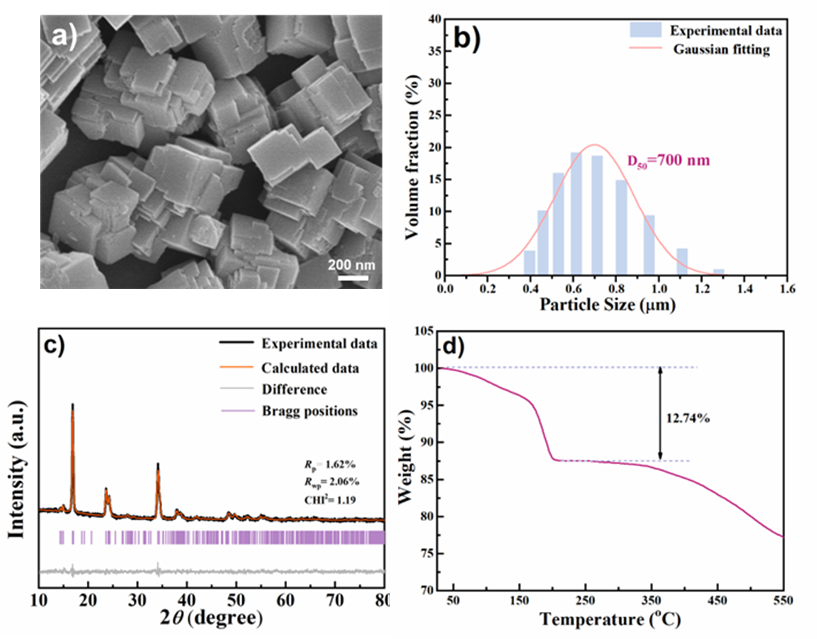


**Figure S3.** a) Morphology, b) particle size distribution, c) Rietveld refined XRD patterns and d) TG of TW-PB.


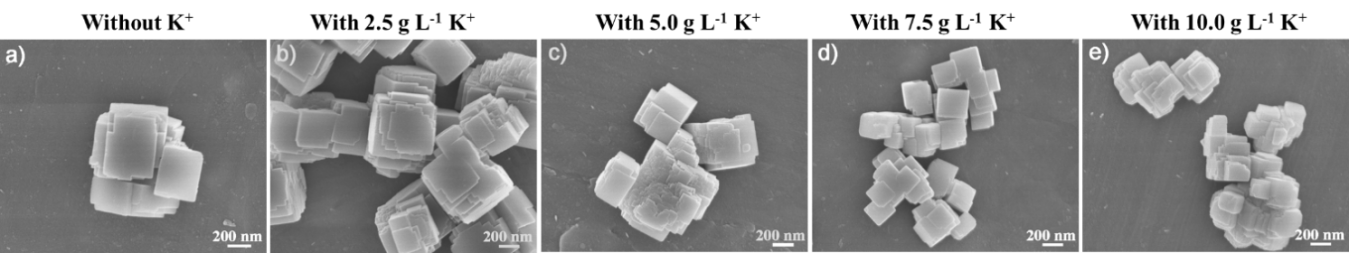


**Figure S4.** Morphology variationof PB with different K^+^ concentrations during synthesis.


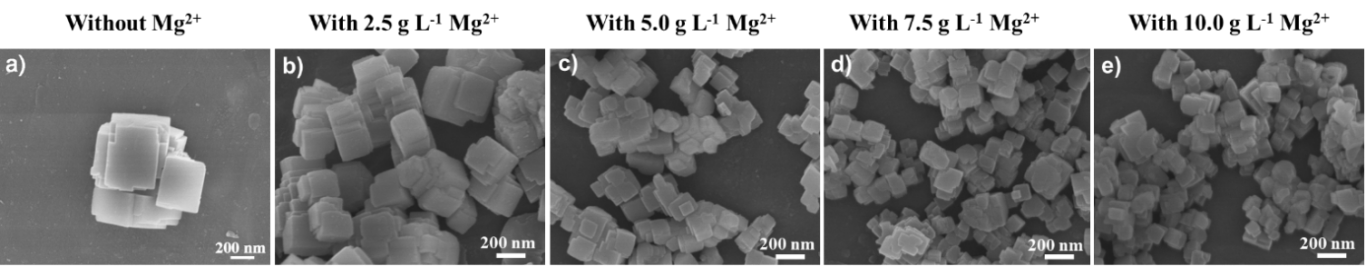


**Figure S5.** Morphology variationof PB with different Mg^2+^ concentrations during synthesis.







**Figure S6.** XRD patternsof PB samples synthesized with different concentrations of (a) K^+^ and (b) Mg^2+^.


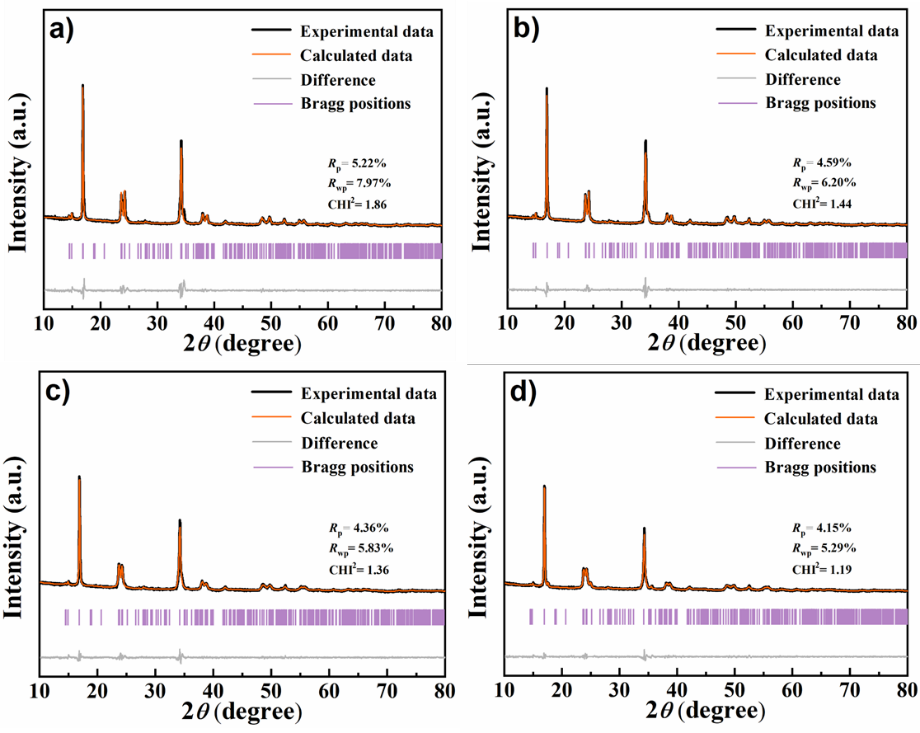


**Figure S7.** Rietveld refined XRD patterns of the PB samples synthesized with gradient K^+^ concentration. (a) 2.5 g L^‒1^ K^+^, (b) 5.0 g L^‒1^ K^+^, (c) 7.5 g L^‒1^ K^+^ and (d) 10.0 g L^‒1^ K^+^.


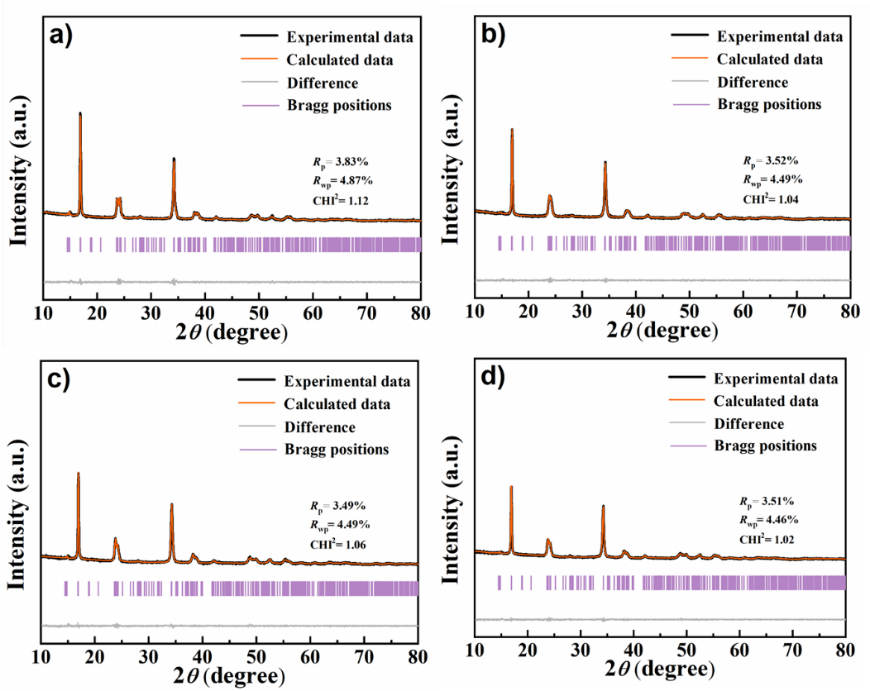


**Figure S8.** Rietveld refined XRD patterns of the PB samples synthesized with gradient Mg^2+^ concentration. (a) 2.5 g L^‒1^ Mg^2+^, (b) 5.0 g L^‒1^ Mg^2+^, (c) 7.5 g L^‒1^ Mg^2+^ and (d) 10.0 g L^‒1^ Mg^2+^.


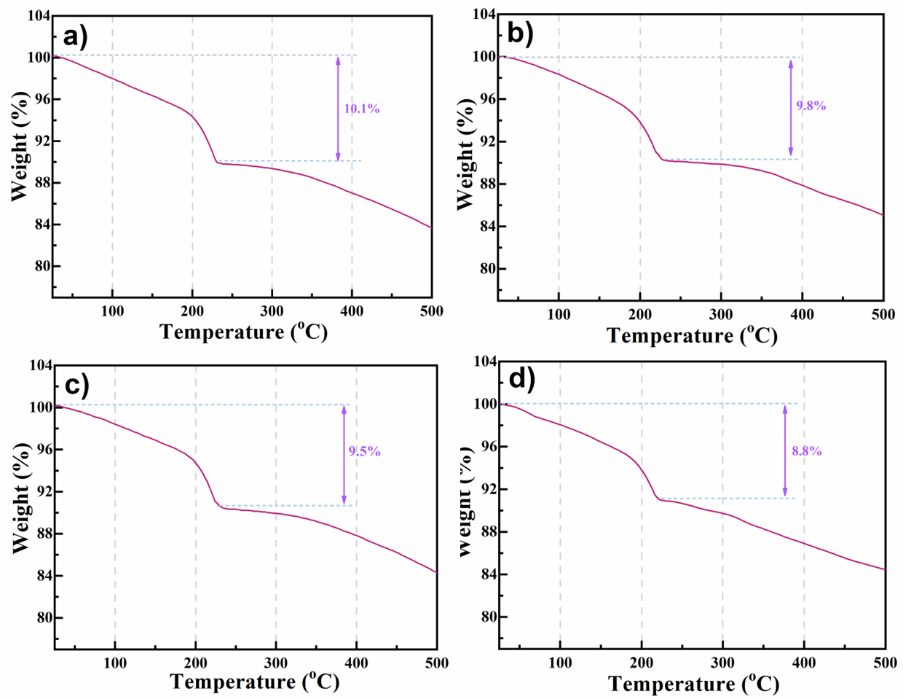


**Figure S9.** TG results of the PB synthesized with gradient K^+^ concentration. (a) 2.5 g L^‒1^ K^+^, (b) 5.0 g L^‒1^ K^+^, (c) 7.5 g L^‒1^ K^+^ and (d) 10.0 g L^‒1^ K^+^.


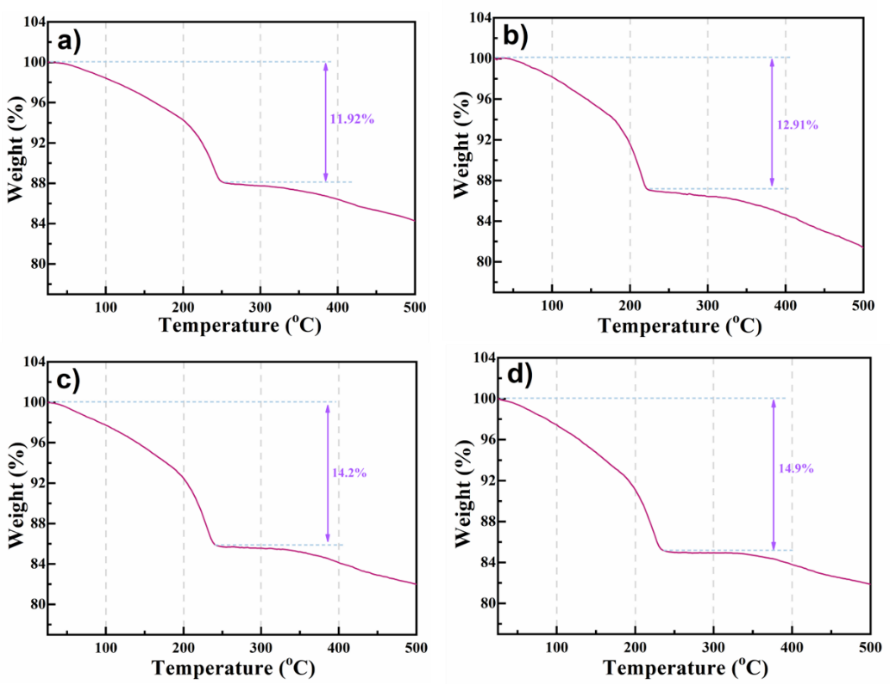


**Figure S10.** TG results of the PB samples synthesized with gradient Mg^2+^ concentration. (a) 2.5 g L^‒1^ Mg^2+^, (b) 5.0 g L^‒1^ Mg^2+^, (c) 7.5 g L^‒1^ Mg^2+^ and (d) 10.0 g L^‒1^ Mg^2+^.





**Figure S11**. Electrochemical performance of DW-PB. a) Voltage curves in the initial three cycles at 0.1 C. b) Discharge curves and c) discharge capacity from 0.1 C to 20 C. d) Cycling stability at 5 C.





**Figure S12**. Electrochemical performance of LW-PB. a) Voltage curves in the initial three cycles at 0.1 C. b) Discharge curves and c) discharge capacity from 0.1 C to 20 C. d) Cycling stability at 5 C.

**

**

**Figure S13**. Electrochemical performance of TW-PB. (a) Voltage curves in the initial three cycles at 0.1 C. b) Discharge curves and c) discharge capacity from 0.1 C to 20 C. d) Cycling stability at 5 C.





**Figure S14**. Electrochemical performance of PB synthesized by prepared Mg^2+^, K^+^ solution. (a) Voltage curves in the initial three cycles at 0.1 C. b) Discharge curves and c) discharge capacity from 0.1 C to 20 C. d) Cycling stability at 5 C.





**Figure S15**. Electrochemical performance of SW-PB washed by sea water. (a) Voltage curves in the initial three cycles at 0.1 C. b) Discharge curves and c) discharge capacity from 0.1 C to 20 C. d) Cycling stability at 5 C.





**Figure S16**. Electrochemical performance of PB synthesized in 0.5 g L^‒1^ Mg^2+^ + 0.3 g L^‒1^ K^+^ solution. a) Voltage curves in the initial three cycles at 0.1 C. b) Discharge curves and c) discharge capacity from 0.1 C to 20 C. d) Cycling stability at 5 C.





**Figure S17**. Electrochemical performance of PB synthesized in 0.75 g L^‒1^ Mg^2+^ + 0.45 g L^‒1^ K^+^ solution. a) Voltage curves in the initial three cycles at 0.1 C. b) Discharge curves and c) discharge capacity from 0.1 C to 20 C. d) Cycling stability at 5 C.





**Figure S18**. Electrochemical performance of PB synthesized in 1.25 g L^‒1^ Mg^2+^ + 0.75 g L^‒1^ K^+^ solution. a) Voltage curves in the initial three cycles at 0.1 C. b) Discharge curves and c) discharge capacity from 0.1 C to 20 C. d) Cycling stability at 5 C.

**

**

**Figure S19**. Electrochemical performance of PB synthesized in 1.5 g L^‒1^ Mg^2+^ + 0.9 g L^‒1^ K^+^ solution. a) Voltage curves in the initial three cycles at 0.1 C. b) Discharge curves and c) discharge capacity from 0.1 C to 20 C. d) Cycling stability at 5 C.


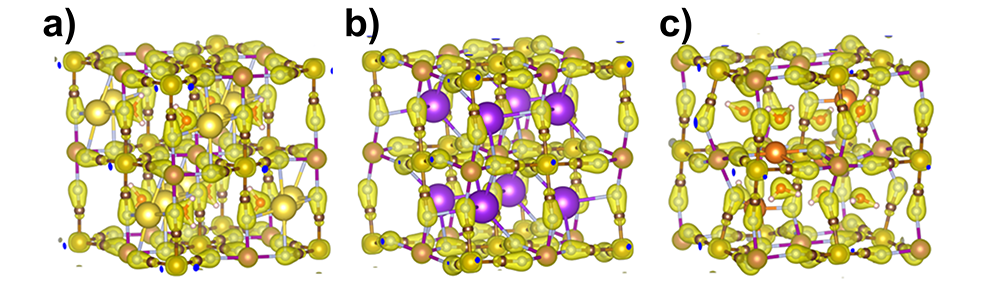


**Figure S20.** The electronic structures of (a) Na-PB, (b) Mg-PB and (c) K-PB frameworks.


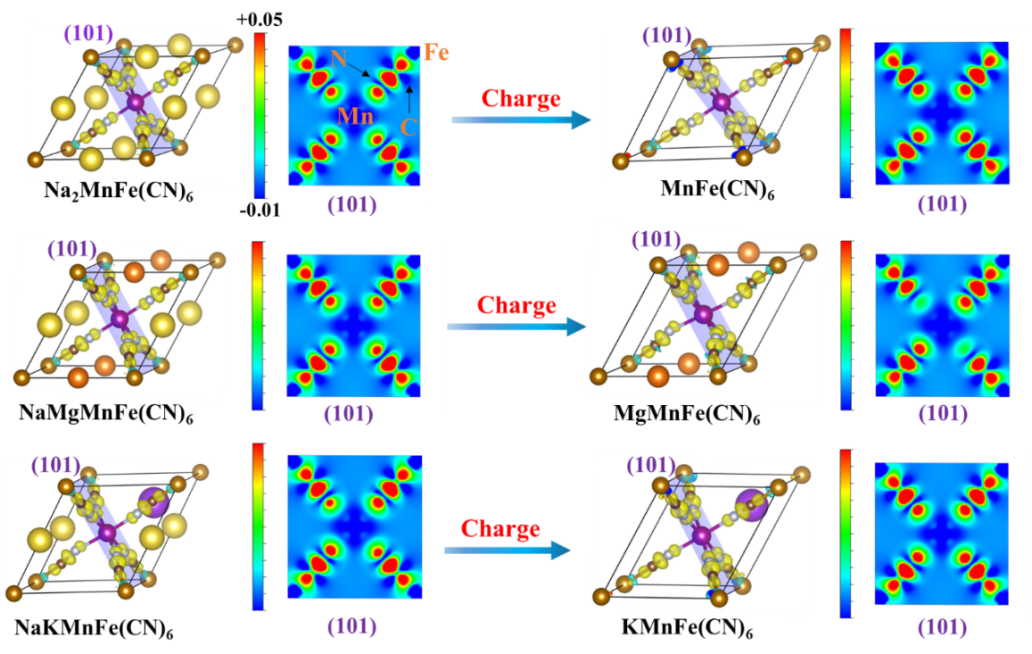


**Figure S21.** Charge density difference of (a) Na-PB, (b) Mg^2+^ doped Na-PB and (c) K^+^ doped Na-PB during Na extraction process. Note that the significant changed charge projection diagram in MgMnFe(CN)_6_ structure originates from the structural distortion of strong Mg‒N Coulombic attraction.


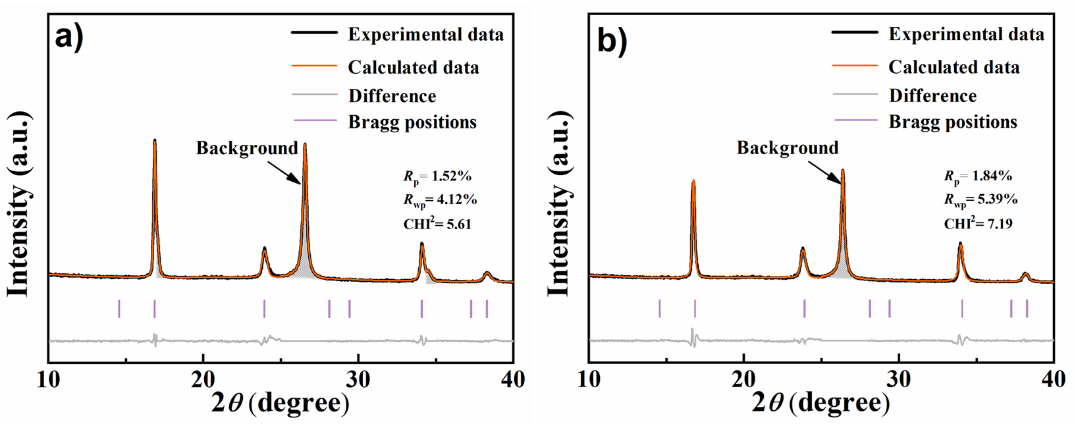


**Figure S22.** XRD Rietveld refined patterns of (a) DW-PB and (b) SW-PB at 4 V. Noting that the original Rietveld refined data exclude the gray area.


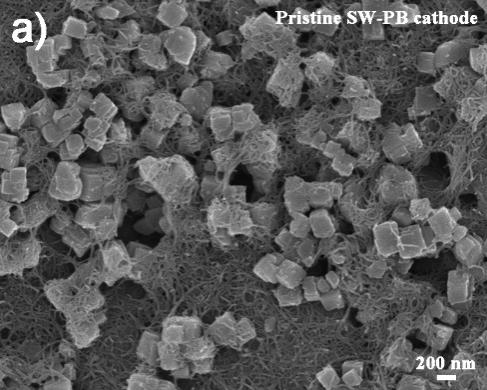

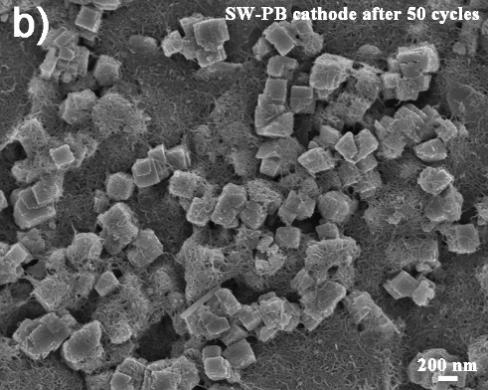


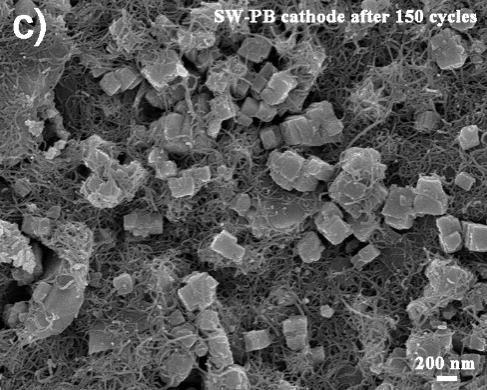

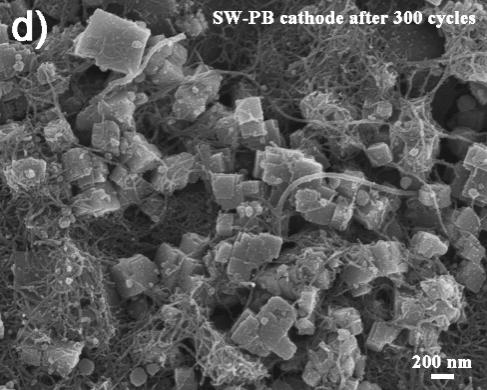


**Figure S23.** Morphology variation of the SW-PB cathodes after different cycles: (a) 0 cycles, (b) 50 cycles, (c) 150 cycles and (d) 300 cycles.


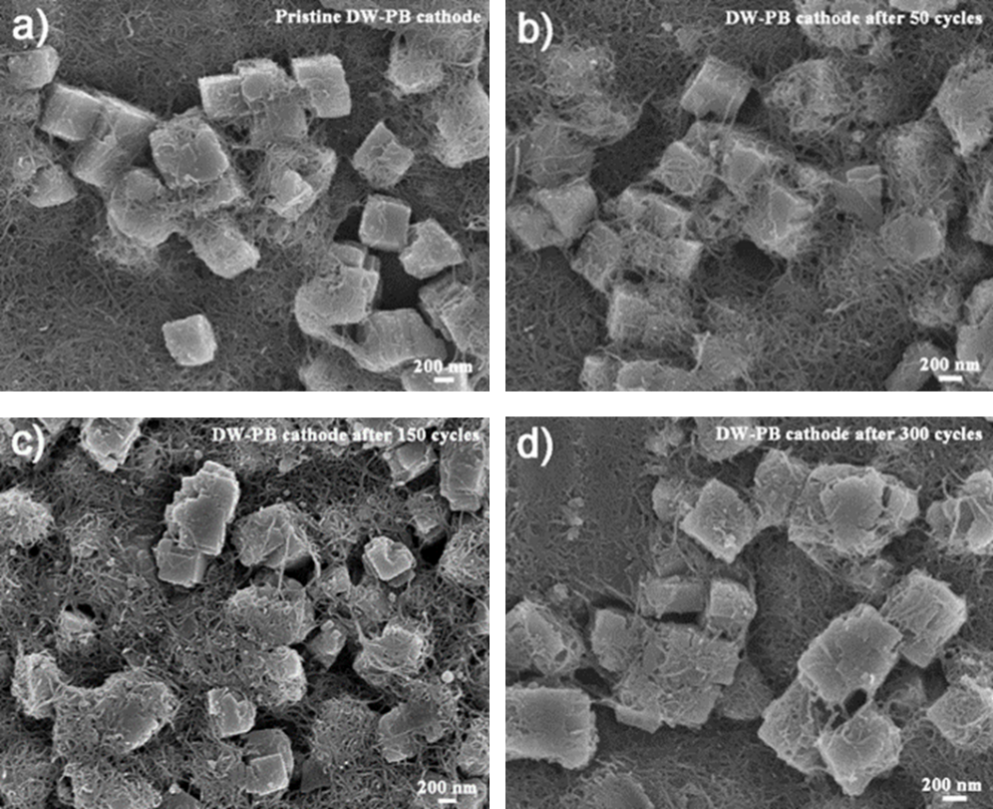


**Figure S24.** Morphology variation of the DW-PB cathodes after different cycles: (a) 0 cycles, (b) 50 cycles, (c) 150 cycles and (d) 300 cycles.


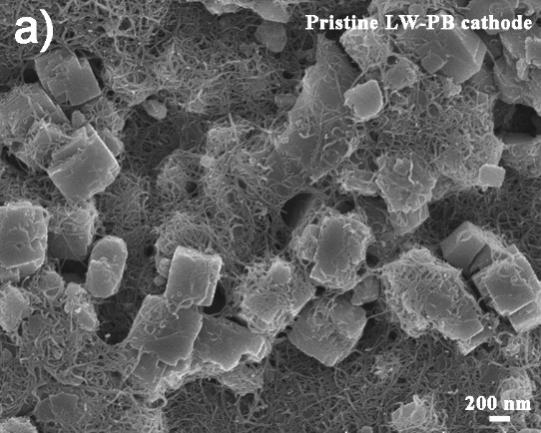

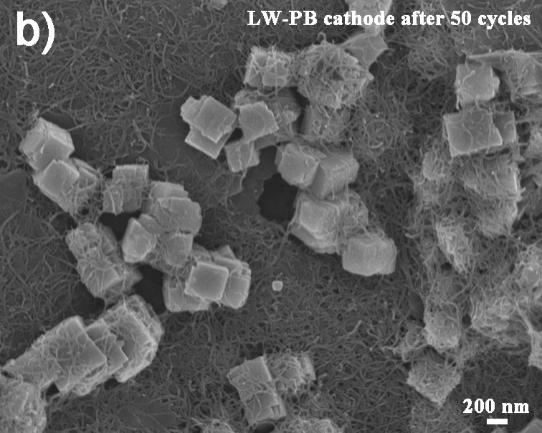


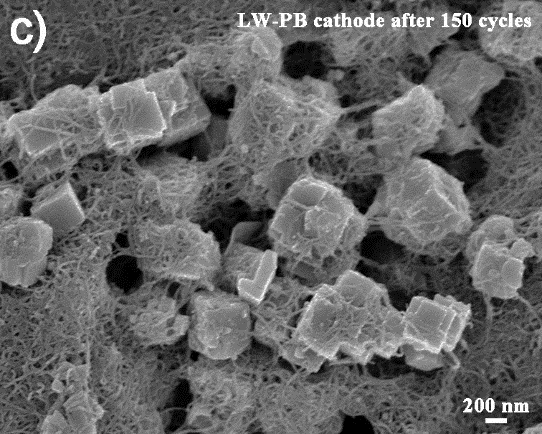

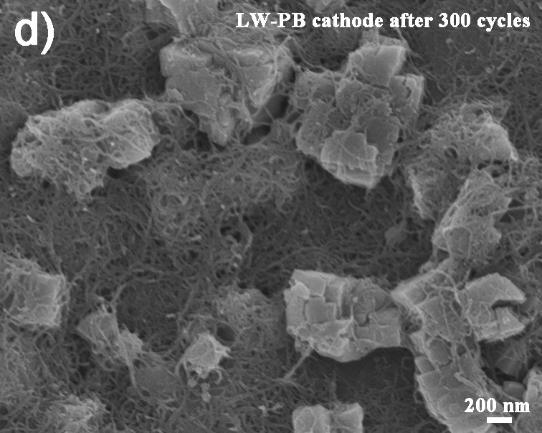


**Figure S25.** Morphology variation of the LW-PB cathode after different cycles: (a) 0 cycles, (b) 50 cycles, (c) 150 cycles and (d) 300 cycles.


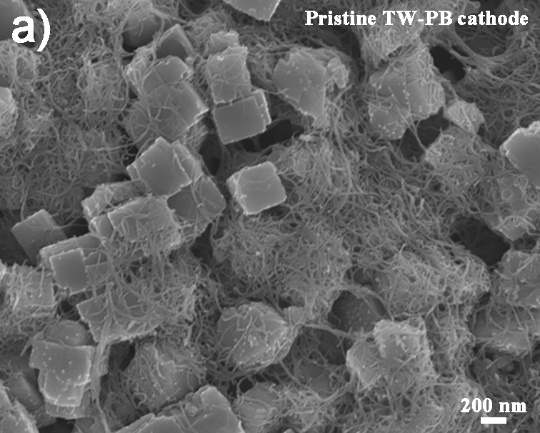

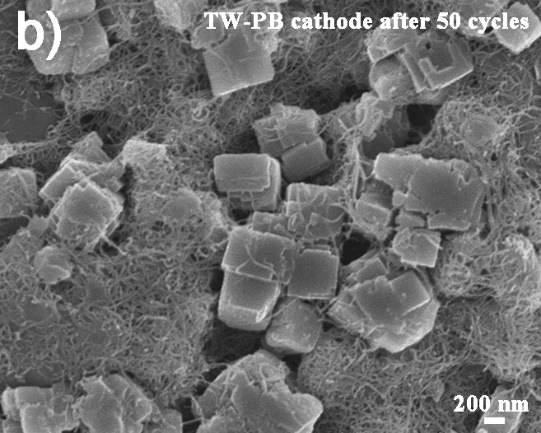


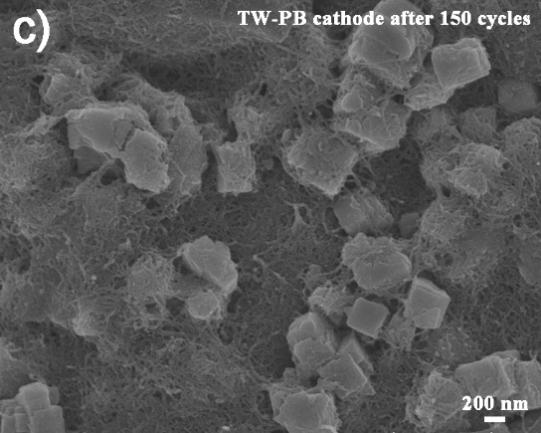

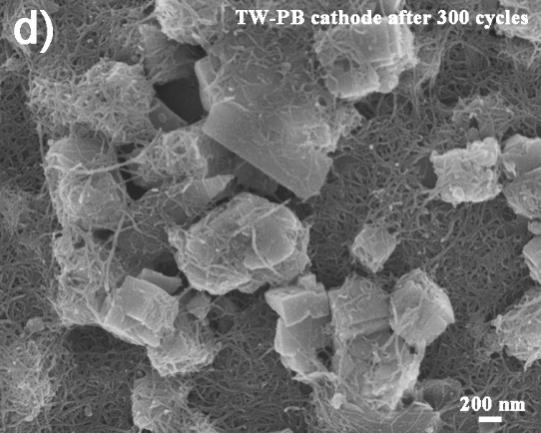


**Figure S26.** Morphology variation of the TW-PB cathodes after different cycles. (a) 0 cycles, (b) 50 cycles, (c) 150 cycles and (d) 300 cycles.


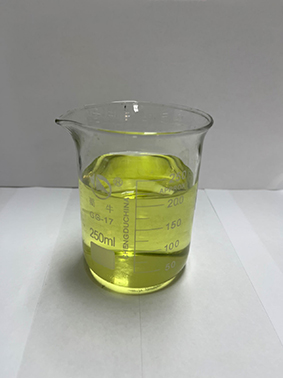


**Figure S27.** Image of the waste water after synthesis of DW-PB.

**
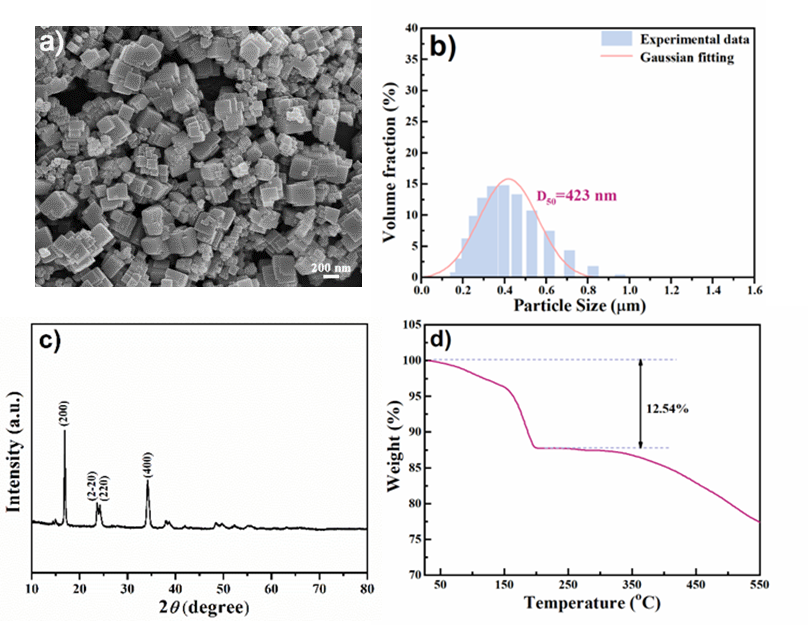
**

**Figure S28.** a) Morphology, b) particle size distribution, c) XRD patterns and d) TG of PB from the waste water.





**Figure S29**. Electrochemical performance of the waste water PB. a) Voltage profiles in the initial three cycles at 0.1 C. b) Discharge curves and c) discharge capacity from 0.1 C to 20 C. d) Cycling stability at 5 C.


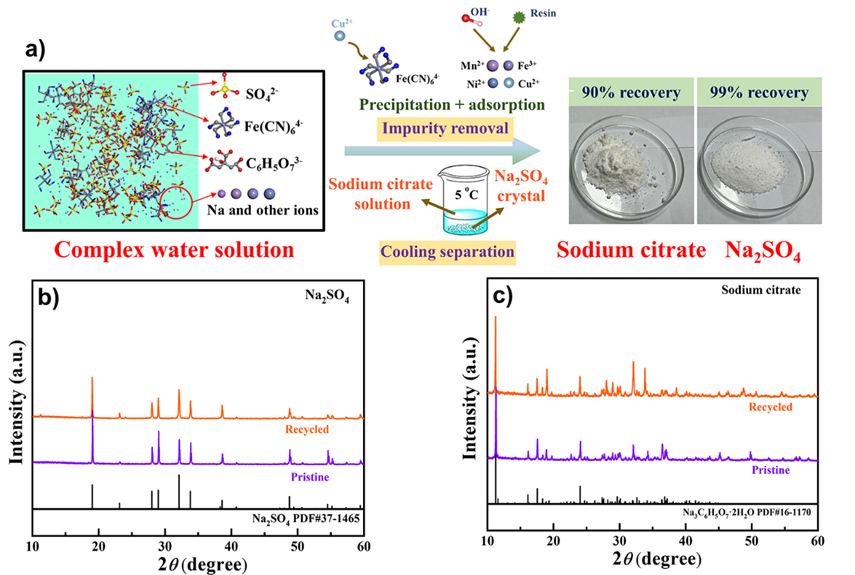







**Figure S30.** a) Schematic diagram of regeneration of sodium citrate and Na_2_SO_4_ from the waste water. XRD patterns of the pristine and the recycled salts of b) Na_2_SO_4_ and c) sodium citrate.

**
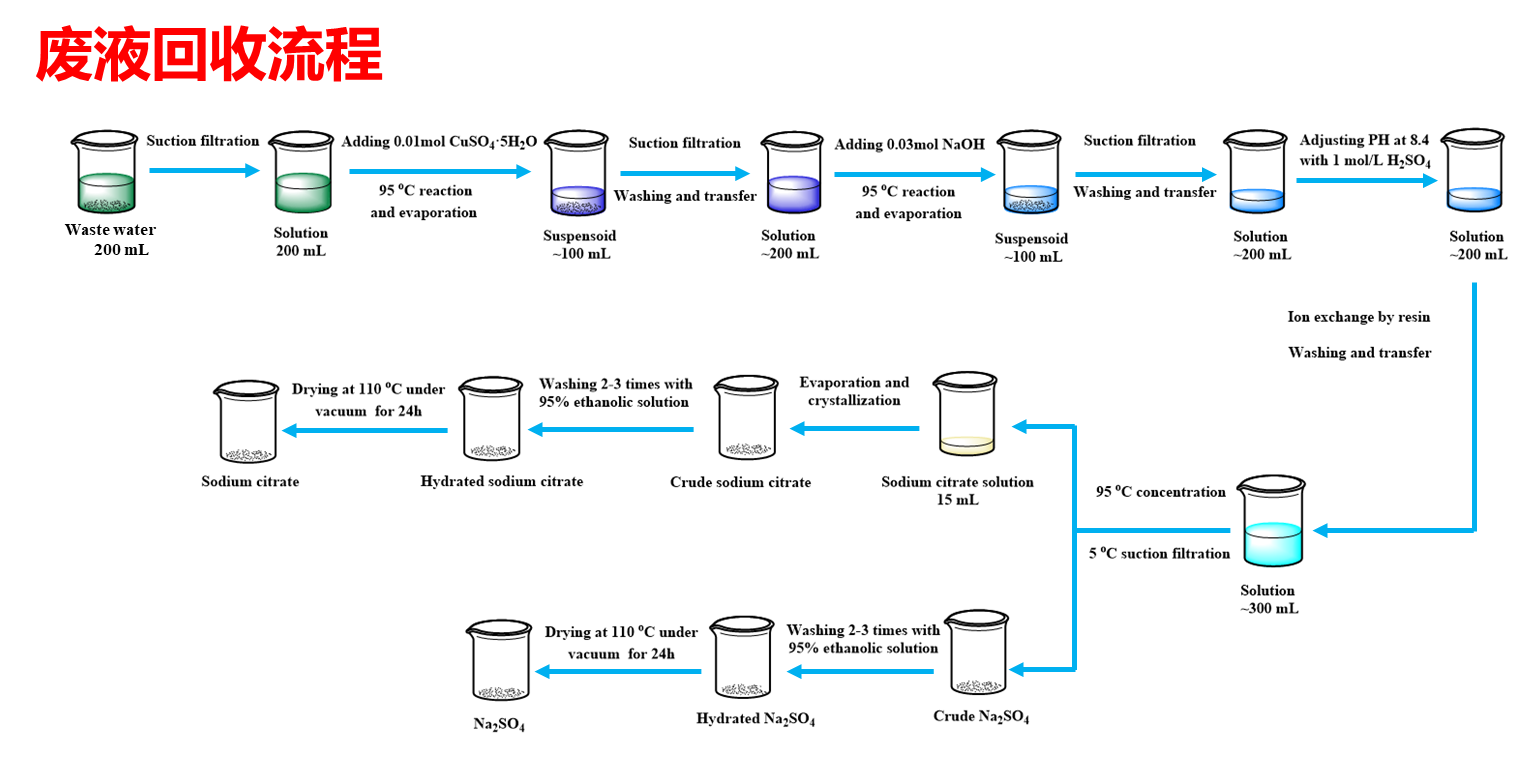
**

**Figure S31.** Detailed purification and sodium salts recycling process of the waste water.


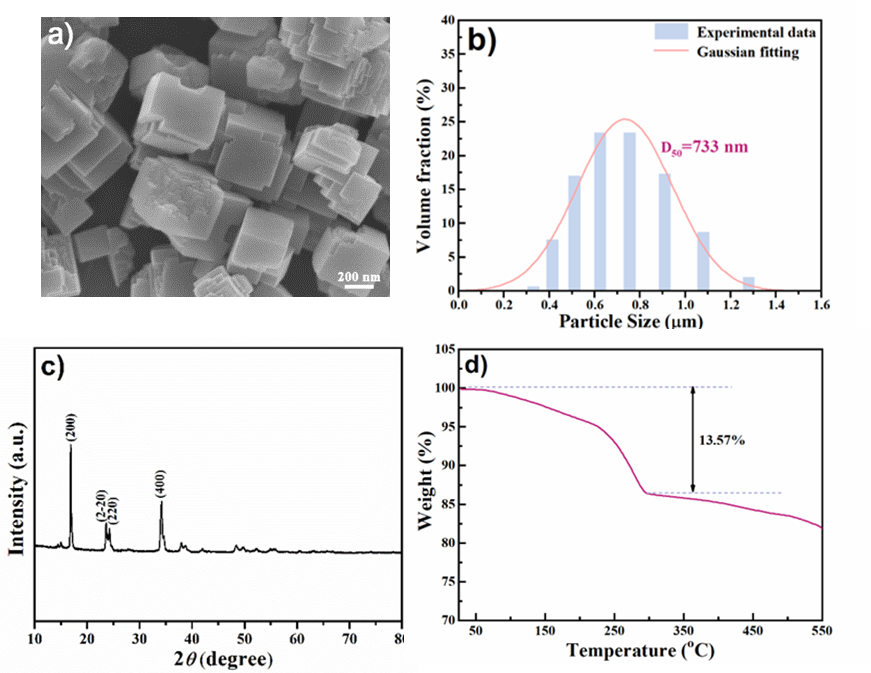


**Figure S32.** a) Morphology, b) particle size distribution, c) XRD patterns and d) TG of the PB from the recycled sodium salts.

**Table S1**. Lattice parameters of the PB materials prepared in different water.

| **Lattice parameters** | **PB materials** | | | |
| --- | --- | --- | --- | --- |
|  | **DW-PB** | **SW-PB** | **TW-PB** | **LW-PB** |
| *a* / Å | 10.5368 | 10.5304 | 10.5409 | 10.5424 |
| *b* / Å | 7.5492 | 7.5426 | 7.5498 | 7.5475 |
| *c* / Å | 7.3305 | 7.3181 | 7.3310 | 7.3228 |
| *α */ ° | 90.000 | 90.000 | 90.000 | 90.000 |
| *β */ ° | 91.671 | 91.240 | 91.541 | 91.686 |
| *γ */ ° | 90.000 | 90.000 | 90.000 | 90.000 |
| *V /* Å^3^ | 582.9 | 581.1 | 583.2 | 582.4 |

**Table S2**. Element composition of the PB materials prepared in different water (mg L^‒1^).

| **Sample** | **Na** | **Mg** | **K** | **Ca** | **Mn** | **Fe** | **Ni** |
| --- | --- | --- | --- | --- | --- | --- | --- |
| DW-PB | 11.88 | ND | ND | ND | 10.32 | 18.35 | 2.71 |
| SW-PB | 11.71 | 0.29 | 0.16 | ND | 10.35 | 19.05 | 2.86 |
| LW-PB | 11.70 | ND | ND | ND | 10.53 | 18.62 | 2.55 |
| TW-PB | 11.69 | ND | ND | ND | 10.69 | 18.01 | 2.74 |

**Table S3.** Chemical formulas of the PB materials synthesized in different water.

| **Sample** | **Chemical formulas** |
| --- | --- |
| DW-PB | Na_1.79_Mn_0.65_Fe_0.19_Ni_0.16_[Fe(CN)_6_]_0.93_·2.23H_2_O |
| SW-PB | Na_1.72_Mg_0.040_ K_0.013_Mn_0.64_Fe_0.20_Ni_0.16_[Fe(CN)_6_]_0.95_·2.01H_2_O |
| LW-PB | Na_1.73_Mn_0.65_Fe_0.20_Ni_0.15_[Fe(CN)_6_]_0.93_·2.19H_2_O |
| TW-PB | Na_1.74_Mn_0.66_Fe_0.18_Ni_0.16_[Fe(CN)_6_]_0.94_·2.39H_2_O |

**Table S4**. Lattice parameters of the PB materials prepared by gradient K^+^ concentration.

| **Lattice parameters** | **Without** | **2.5 g L^‒1^** | **5.0 g L^‒1^** | **7.5 g L^‒1^** | **10.0 g L^‒1^** |
| --- | --- | --- | --- | --- | --- |
| *a* / Å | 10.5368 | 10.4856 | 10.4824 | 10.4773 | 10.4383 |
| *b* / Å | 7.5492 | 7.5305 | 7.5213 | 7.5076 | 7.4698 |
| *c* / Å | 7.3305 | 7.3110 | 7.3169 | 7.3103 | 7.3122 |
| *α */ ° | 90.000 | 90.000 | 90.000 | 90.000 | 90.000 |
| *β */ ° | 91.671 | 91.599 | 91.442 | 90.973 | 90.860 |
| *γ */ ° | 90.000 | 90.000 | 90.000 | 90.000 | 90.000 |
| *V /* Å^3^ | 582.9 | 577.1 | 576.7 | 574.9 | 570.1 |

**Table S5**. Lattice parameters of the PB materials prepared by gradient Mg^2+^ concentration.

| **Lattice parameters** | **Without** | **2.5 g L^‒1^** | **5.0 g L^‒1^** | **7.5 g L^‒1^** | **10.0 g L^‒1^** |
| --- | --- | --- | --- | --- | --- |
| *a* / Å | 10.5368 | 10.5278 | 10.4569 | 10.4645 | 10.4626 |
| *b* / Å | 7.5492 | 7.5365 | 7.4934 | 7.4886 | 7.5075 |
| *c* / Å | 7.3305 | 7.3229 | 7.2874 | 7.2905 | 7.2929 |
| *α */ ° | 90.000 | 90.000 | 90.000 | 90.000 | 90.000 |
| *β */ ° | 91.671 | 91.369 | 90.833 | 90.894 | 90.798 |
| *γ */ ° | 90.000 | 90.000 | 90.000 | 90.000 | 90.000 |
| *V /* Å^3^ | 582.9 | 580.9 | 571.0 | 571.3 | 572.8 |

**Table S6.** Related ion concentration in different water (mg L^‒1^).

| **Sample** | **Na** | **Mg** | **K** | **Ca** | **Al** | **Mn** | **Fe** | **Ni** |
| --- | --- | --- | --- | --- | --- | --- | --- | --- |
| DI water | 0.00 | 0.00 | 0.00 | 0.00 | 0.00 | 0.00 | 0.00 | 0.00 |
| Sea water | 6818.72 | 1001.29 | 582.89 | 315.55 | 0.04 | 0.00 | 0.01 | 0.01 |
| Lake water | 6.24 | 1.88 | 0.93 | 9.91 | 0.01 | 0.00 | 0.00 | 0.00 |
| Tap water | 5.52 | 2.19 | 1.62 | 12.95 | 0.05 | 0.00 | 0.02 | 0.01 |

**Table S7.** Intercalation energy of ions into MnFe(CN)_6_ framework.

| **Ion** | **Radius (Å)** | ***E*_b1_** | | | |
| --- | --- | --- | --- | --- | --- |
|  |  | **24d** | **32f(c)** | **32f(n)** | **8c** |
| Na^+^ | 1.02 | **-3.64** | -1.82 | -2.34 | -1.89 |
| K^+^ | 1.38 | -3.51 | -0.37 | -1.14 | **-4.09** |
| Mg^2+^ | 0.72 | **-4.09** | -2.22 | -2.89 | -0.58 |
| Ca^2+^ | 1.00 | **-5.71** | -3.20 | -3.22 | -5.10 |

**Table S8.** Intercalation energy of ions into NaMnFe(CN)_6_ framework.

| **Ion** | **Radius (Å)** | ***E*_b2_** | | | |
| --- | --- | --- | --- | --- | --- |
|  |  | **24d** | **32f(c)** | **32f(n)** | **8c** |
| Na^+^ | 1.02 | **-2.42** | 0.13 | -1.44 | -1.29 |
| K^+^ | 1.38 | **-2.29** | 2.35 | -0.19 | **-2.30** |
| Mg^2+^ | 0.72 | 0.03 | 1.66 | 0.48 | 1.11 |
| Ca^2+^ | 1.00 | **-1.48** | 1.79 | 2.02 | 0.36 |

**Table S9**. Element composition of the PB materials at charge state (mg L^‒1^).

| **Sample** | **Pristine state** | | | **Charge state** | | |
| --- | --- | --- | --- | --- | --- | --- |
|  | **Na** | **Mg** | **K** | **Na** | **Mg** | **K** |
| DW-PB | 11.88 | ND | ND | 4.36 | ND | ND |
| SW-PB | 11.71 | **0.29** | **0.16** | 4.13 | **0.28** | **0.16** |
| LW-PB | 11.70 | ND | ND | 4.05 | ND | ND |
| TW-PB | 11.69 | ND | ND | 4.14 | ND | ND |

Note: The Na, Mg, K concentration at charge state is converted based on same Fe concentration at pristine state.

**Table S10.** Impurity influence on the electrochemical performance of PB.

| **Sample** | **Capacity**  **(mAh g^-1^, 0.1 C)** | **Rate capability**  **(mAh g^-1^, 20C)** | **Cycling stability**  **(retention@cycles, 5C)** |
| --- | --- | --- | --- |
| Sea water synthesis / DI water washing | 115.8 | 104.7 | 97.7%@250, 5C |
| Comparable Mg^2+^, K^+^ synthesis  （DI water synthesis / washing) | 114.7 | 104.9 | 96.4%@250, 5C |
| Sea water synthesis / Sea water washing | 117.3 | 104.5 | 96.2%@250, 5C  89.5%@450, 5C |

**Table S11.** Chemical formulas of PB materials prepared in different Mg^2+^, K^+^ solutions.

| **Sample** | **Mg^2+^, K^+^ concentration** | **Chemical formulas** |
| --- | --- | --- |
| Sample 1 | 0.5 g L^‒1^ Mg^2+^ + 0.3 g L^‒1^ K^+^ | Na_1.72_Mg_0.011_K_0.006_Mn_0.63_Fe_0.20_Ni_0.17_[Fe(CN)_6_]_0.94_ |
| Sample 2 | 0.75 g L^‒1^ Mg^2+^ + 0.45 g L^‒1^ K^+^ | Na_1.71_Mg_0.027_K_0.011_Mn_0.64_Fe_0.20_Ni_0.16_[Fe(CN)_6_]_0.97_ |
| Sample 3 | 1.25 g L^‒1^ Mg^2+^ + 0.75 g L^‒1^ K^+^ | Na_1.73_Mg_0.053_K_0.033_Mn_0.63_Fe_0.20_Ni_0.17_[Fe(CN)_6_]_0.97_ |
| Sample 4 | 1.5 g L^‒1^ Mg^2+^ + 0.9 g L^‒1^ K^+^ | Na_1.66_Mg_0.087_K_0.049_Mn_0.64_Fe_0.20_Ni_0.16_[Fe(CN)_6_]_0.97_ |

**Table S12.** Electrochemical performance of the PB materials with gradient Mg^2+^, K^+^ doping.

| **Sample** | **Capacity**  **(mAh g^‒1^, 0.1 C)** | **Rate capacity**  **(mAh g^‒1^, 20C)** | **Cycling stability**  **(retention@cycles, 5C)** |
| --- | --- | --- | --- |
| Sample 1 | 118.9 | 106.3 | 94.8%@250, 5C |
| Sample 2 | 117.2 | 103.3 | 95.9%@250, 5C |
| Sample 3 | 115.8 | 103.0 | 95.9%@250, 5C |
| Sample 4 | 110.9 | 91.7 | 94.8%@250, 5C |

Note: the capacity retention for SW-PB after 250 cycles is 97.7% and the capacity retention for DW-PB after 250 cycles is 93.9%.

**Table S13.** Calculated charge transfer (e) from Na, Mg, K to N atom for Na-PB, Mg^-^PB and K-PB.

| **Structure** | **Charge transfer value (e)** |
| --- | --- |
| Na-PB | 0.85 |
| Mg-PB | 1.69 |
| K-PB | 0.94 |

**Table S14.** Elements dissolution analysis of Mn, Fe, Ni for SW-PB and DW-PB (mg L^‒1^).

| **State** | **SW-PB** | | | **DW-PB** | | |  |
| --- | --- | --- | --- | --- | --- | --- | --- |
|  | **Mn** | **Fe** | **Ni** | **Mn** | **Fe** | **Ni** | |
| Pristine | 10.35 | 19.05 | 2.86 | 10.71 | 19.05 | 2.81 | |
| After 300 cycles | 10.09 | 19.05 | 2.74 | 9.86 | 19.05 | 2.63 | |

Note: The Mn, Ni concentration after cycling is converted based on same Fe concentration at pristine state.

**Table S15.** Bader charge variation (e) on N atom coordinated to central Mn atom for Na-PB during charge/discharge process.

| **Label** | **Discharge state** | **Charge state** |
| --- | --- | --- |
| N1 | 6.44 | 6.21 |
| N2 | 6.44 | 6.21 |
| N3 | 6.43 | 6.20 |
| N4 | 6.43 | 6.20 |
| N5 | 6.35 | 6.21 |
| N6 | 6.35 | 6.21 |

**Table S16.** Bader charge variation (e) on N atom coordinated to central Mn atom for Mg^2+^ doped Na-PB after Na extraction.

| **Label** | **Discharge state** | **Charge state** |
| --- | --- | --- |
| N1 | 6.46 | 6.49 |
| N2 | 6.46 | 6.49 |
| N3 | 6.43 | 6.48 |
| N4 | 6.43 | 6.48 |
| N5 | 6.43 | 6.37 |
| N6 | 6.43 | 6.37 |

**Table S17.** Bader charge variation (e) on N atom coordinated to central Mn atom for K^+^ doped Na-PB after Na extraction.

| **Label** | **Discharge state** | **Charge state** |
| --- | --- | --- |
| N1 | 6.44 | 6.31 |
| N2 | 6.47 | 6.30 |
| N3 | 6.41 | 6.29 |
| N4 | 6.44 | 6.36 |
| N5 | 6.36 | 6.30 |
| N6 | 6.36 | 6.31 |

**Table S18.** Lattice parameters variation for SW-PB and DW-PB after charge to 4 V.

| **Lattice parameters** | **SW-PB** | | **DW-PB** | |
| --- | --- | --- | --- | --- |
|  | **Pristine state** | **Charge state** | **Pristine state** | **Charge state** |
| *a* / Å | 10.5304 | 7.430 | 10.5368 | 7.420 |
| *b* / Å | 7.5426 | 7.430 | 7.5492 | 7.420 |
| *c* / Å | 7.3181 | 7.430 | 7.3305 | 7.420 |
| *α */ ° | 90.000 | 90.000 | 90.000 | 90.000 |
| *β */ ° | 91.240 | 90.000 | 91.671 | 90.000 |
| *γ */ ° | 90.000 | 90.000 | 90.000 | 90.000 |
| *V /* Å^3^ | 581.1 | 580.1 | 582.9 | 579.4 |
| *ΔV%* | 0.17% | | 0.60% | |

**Table S19.** Element composition of the PB prepared by waste water and recycled salts (mg L^‒1^).

| **Sample** | **Na** | **Mn** | **Fe** | **Ni** | **Cu** |
| --- | --- | --- | --- | --- | --- |
| Waste water | 13.62 | 11.62 | 22.72 | 4.74 | / |
| Recycled salts | 16.1 | 15.0 | 26.2 | 4.14 | ND |

**Table S20.** Chemical formulas of the waste water sample and recycled salts sample.

| **Sample** | **Chemical formulas** |
| --- | --- |
| Waste water | Na_1.61_Mn_0.57_Fe_0.20_Ni_0.23_[Fe(CN)_6_]_0.90_·2.26H_2_O |
| Recycled salts | Na_1.64_Mn_0.65_Fe_0.19_Ni_0.16_[Fe(CN)_6_]_0.91_·2.49H_2_O |

**Table S21.** Impurity ions concentration in the waste water and recycled salts solution (mg L^‒1^).

| **water** | **Cu** | **Mn** | **Fe** | **Ni** |
| --- | --- | --- | --- | --- |
| Waste water | ND | 14.8 | 713.8 | 326.1 |
| Recycled salts solution | 1.2 | 0.04 | 0.2 | 0.01 |

**Table S22**. Lattice parameters comparison of DW-PB and recycled salts PB.

| **Lattice parameters** | **DW-PB** | **Recycled salts PB** |
| --- | --- | --- |
| *a* / Å | 10.5368 | 10.5323 |
| *b* / Å | 7.5492 | 7.5409 |
| *c* / Å | 7.3305 | 7.3266 |
| *α */ ° | 90.000 | 90.000 |
| *β */ ° | 91.671 | 91.559 |
| *γ */ ° | 90.000 | 90.000 |
| *V /* Å^3^ | 582.9 | 581.7 |

**Table S23.** Comparison of the electrochemical performance of the PB materials in this work.

| **Sample** | **Capacity**  **(mAh g^‒1^, 0.1 C)** | **Capacity**  **(mAh g^‒1^, 5 C)** | **Capacity**  **(mAh g^‒1^, 20 C)** | **Cycling stability**  **(retention@cycles,5C)** |
| --- | --- | --- | --- | --- |
| **SW-PB** | **115.8** | **108.2** | **104.7** | **93.9%@500, 5C**  **85.0%@1000, 5C**  **80.1%@2400, 5C** |
| Recycled salts PB | 116.7 | 108.5 | 104.6 | 89.6%@500, 5C |
| DW-PB | 121.5 | 116.3 | 111.7 | 88.6%@500, 5C |
| LW-PB | 118.5 | 112.5 | 109.5 | 88.4%@500, 5C |
| TW-PB | 118.5 | 111.7 | 108.4 | 87.6%@500, 5C |
| Waste water PB | 115.5 | 108.7 | 102.4 | 82.8%@500, 5C |

**Table S24.** The production costs for 1kg PB preparation.

(The regents’ prices are from the reagent platform of Sinopharm Chemical Reagent Co., Ltd.)

| **Regents** | **Purity** | **Price ($)** | **Consumption (g)** | | **Costs ($)** |
| --- | --- | --- | --- | --- | --- |
| Na_3_C_6_H_5_O_7_·2H_2_O | AR, ≥99.0% | 156.5/25kg | | 3919.6 | **24.5** |
| Na_2_SO_4_ | AR, ≥99.0% | 61.6/25kg | | 9165.7 | **22.6** |
| MnSO_4_·H_2_O | AR, ≥98.0% | 10.6/1kg | | 339.9 | 3.6 |
| NiSO_4_·6H_2_O | AR, ≥99.0% | 22.9/1kg | | 176.6 | 4.0 |
| FeSO_4_·7H_2_O | AR, ≥99.0% | 5.3/1kg | | 186.6 | 0.99 |
| Na_4_[Fe(CN)_6_]·10H_2_O | AR, ≥99.0% | 30.1/500g | | 1613.2 | 48.5 |

Note: 1$ = 7.125 RMB.

**Table S25.** Production cost for 1 kg PB with and without sodium salts recycling and reuse.

| **Recycling frequency** | Without recycling | Recycling for  4 times | Recycling for  9 times | Recycling for  99 times |
| --- | --- | --- | --- | --- |
| **Average Cost ($)** | 104.3 | 70.3 | 66.1 | 62.3 |

1. Z. Xu, Dr. Y. Huang, Prof. X. Zhao, Dr. J. Xie

   State Key Laboratory of Silicon and Advanced Semiconductor Materials, School of Materials Science and Engineering, Zhejiang University, Hangzhou 310058, China

   1. mail: xiejian1977@zju.edu.cn

   F. Chen

   Shaanxi Coal Chemical Industry Technology Research Institute, Xi’an 710100, China

   Y. Li, Prof. Y. Lu

   School of Physics, Zhejiang University, Hangzhou 310058, China

   Dr. A. Zhou, Dr. J. Jiang

   Yangtze Delta Region Institute (Huzhou), University of Electronic Science and Technology of China, Huzhou 313000, China

   X. Xu, Dr. J. Tu

   LI-FUN Technology Corporation Limited, Zhuzhou 412000, China

   Dr. B. Pan

   Yuna Technology Corporation Limited, Hangzhou 311121, China

   F. Chen

   Department of Chemistry, Zhejiang University, Hangzhou 310058, China [↑](#footnote-ref-1)
